# Initiation of H1-T6SS dueling between *Pseudomonas aeruginosa*

**DOI:** 10.1128/mbio.00355-24

**Published:** 2024-07-11

**Authors:** M. George, S. Narayanan, A. Tejada-Arranz, A. Plack, M. Basler

**Affiliations:** 1 Biozentrum, University of Basel, Basel, Switzerland; Institut Pasteur, Paris, France

**Keywords:** *Pseudomonas aeruginosa*, T6SS, toxins, bacterial interactions, competition

## Abstract

The Type VI secretion system (T6SS) is a multicomponent apparatus, present in many Gram-negative bacteria, which can inhibit bacterial prey in various ecological niches. *Pseudomonas aeruginosa* assembles one of its three T6SS (H1-T6SS) to respond to attacks from adjacent competing bacteria. Surprisingly, repeated assemblies of the H1-T6SS, termed dueling, were described in a monoculture in the absence of an attacker strain; however, the underlying mechanism was unknown. Here, we explored the role of H2-T6SS of *P. aeruginosa* in triggering H1-T6SS assembly. We show that H2-T6SS inactivation in *P. aeruginosa* causes a significant reduction in H1-T6SS dueling and that H2-T6SS activity directly triggers retaliation by the H1-T6SS. Intraspecific competition experiments revealed that elimination of H2-T6SS in non-immune prey cells conferred protection from H1-T6SS. Moreover, we show that the H1-T6SS response is triggered independently of the characterized lipase effectors of the H2-T6SS, as well as those of *Acinetobacter baylyi* and *Vibrio cholerae*. Our results suggest that H1-T6SS response to H2-T6SS in *P. aeruginosa* can impact intraspecific competition, particularly when the H1-T6SS effector-immunity pairs differ between strains, and could determine the outcome of multistrain colonization.

**IMPORTANCE:**

The opportunistic pathogen *Pseudomonas aeruginosa* harbors three different Type VI secretion systems (H1, H2, and H3-T6SS), which can translocate toxins that can inhibit bacterial competitors or inflict damage to eukaryotic host cells. Unlike the unregulated T6SS assembly in other Gram-negative bacteria, the H1-T6SS in *P. aeruginosa* is precisely assembled as a response to various cell damaging attacks from neighboring bacterial cells. Surprisingly, it was observed that neighboring *P. aeruginosa* cells repeatedly assemble their H1-T6SS toward each other. Mechanisms triggering this “dueling” behavior between sister cells were unknown. In this report, we used a combination of microscopy, genetic and intraspecific competition experiments to show that H2-T6SS initiates H1-T6SS dueling. Our study highlights the interplay between different T6SS clusters in *P. aeruginosa*, which may influence the outcomes of multistrain competition in various ecological settings such as biofilm formation and colonization of cystic fibrosis lungs.

## INTRODUCTION

To colonize a certain niche and to compete with other organisms, dedicated protein targeting and secretion systems are deployed by bacteria to efficiently transport proteins or numerous macromolecules to the external environment (1). The Type VI secretion system (T6SS) in Gram-negative bacteria has recently emerged as one of the crucial players, which facilitate contact-dependent toxin delivery into neighboring cells (2, 3). T6SSs are present in many Gram-negative bacteria including *Pseudomonas aeruginosa*, which is an opportunistic pathogen, capable of causing chronic and acute infections (4). It can mainly infect immunocompromised patients and individuals suffering from cystic fibrosis (CF) (5, 6). *P. aeruginosa* carry three T6SS clusters (H1-, H2-, and H3-T6SS) that encode the core T6SS components. The H1-T6SS is mainly anti-bacterial, whereas H2- and H3-T6SSs are crucial for metal ion transport, internalization into host cells, and bacterial antagonism (7–10).

Genetic clusters of T6SS generally consist of a set of 13 core genes that are conserved in Gram-negative species such as *Escherichia coli*, *Vibrio cholerae*, and *P. aeruginosa* (11). These 13 genes encode cytoplasmic, periplasmic, and membrane proteins. The T6SS functionally resembles a contractile bacteriophage tail (12, 13). It is composed of a cytoplasmic contractile sheath, which is tethered to the cell envelope by a base plate structure and a membrane complex (12, 14–16). The sheath contraction powers the translocation of effectors bound to the T6SS spike/tube components, namely, VgrG, PAAR, and Hcp (2, 3, 17, 18). The contracted sheath is specifically disassembled by the AAA+ ATPase ClpV, which facilitates recycling of sheath subunits for new rounds of T6SS assembly (19–21). T6SS activity comprising sheath polymerization, contraction, and disassembly can be visualized by live-cell fluorescence microscopy (21). Cells secreting anti-bacterial toxins are protected by cognate immunity proteins often encoded immediately downstream of the effector genes (22).

Several mechanisms regulate the T6SS expression and activity in *P. aeruginosa*. The posttranscriptional regulator RsmA negatively regulates the expression of all the three T6SS clusters (23). The RsmA-dependent posttranscriptional repression can be relieved by the removal of RetS, which is a sensor kinase (24). The deletion of *retS* activates the GacS/GacA two-component system, which leads to the expression of two small noncoding RNAs, RsmY and RsmZ. These noncoding RNAs further sequester the T6SS-inhibitory transcriptional regulator RsmA (2, 25, 26). In addition, previous studies showed that the H1-T6SS is posttranslationally controlled by the threonine phosphorylation pathway (TPP) encoded next to the H1-T6SS cluster (2, 27, 28). The TPP components are the TagQRST sensor module, the PpkA kinase that can phosphorylate its substrate, Fha, and its cognate phosphatase, PppA (29, 30). Activation of PpkA by a poorly characterized mechanism dependent on TagQRST results in Fha phosphorylation, which triggers H1-T6SS assembly precisely positioned to the site where *P. aeruginosa* is attacked by a neighboring bacterium (21, 29–31). Apart from T6SS-mediated attacks from competing bacteria, membrane perturbations by T4SS, reagents like EDTA or polymyxin B and extracellular DNA can also stimulate the TagQRST sensor module to trigger H1-T6SS assembly (32, 33). Furthermore, it was shown that *V. cholerae* V52 T6SS effector TseL with phospholipase activity was required and sufficient for triggering a response for the H1-T6SS of *P. aeruginosa* (34). However, another report demonstrated that *Acinetobacter baylyi* ADP1 lacking all known effectors, including a lipase, can also trigger H1-T6SS (35), suggesting that physical puncture may be sufficient under some conditions to elicit an H1-T6SS response. Interestingly, it was observed that pairs of *P. aeruginosa* sister cells engage in spatially and temporally correlated rounds of H1-T6SS assemblies, termed dueling (21, 31). Because H1-T6SS assembly is a result of an external stimulus, such dueling was attributed to accidental H1-T6SS attacks from neighboring sister cells (21).

Here, we reasoned that the assembly of H2-T6SS or H3-T6SS may be responsible for initiating H1-T6SS dueling and indeed show that inactivation of H2-T6SS reduced H1-T6SS dueling. In addition, we show by fluorescence microscopy and intraspecific competition experiments that the H2-T6SS, or the T6SS of *V. cholerae* 2740-80 and *A. baylyi*, can trigger an H1-T6SS response, even in the absence of lipase effectors, suggesting that such effectors are dispensable for triggering retaliation by the H1-T6SS.

## RESULTS

### H1-T6SS assembly rate is increased by cell–cell contact

**Fig 1:**
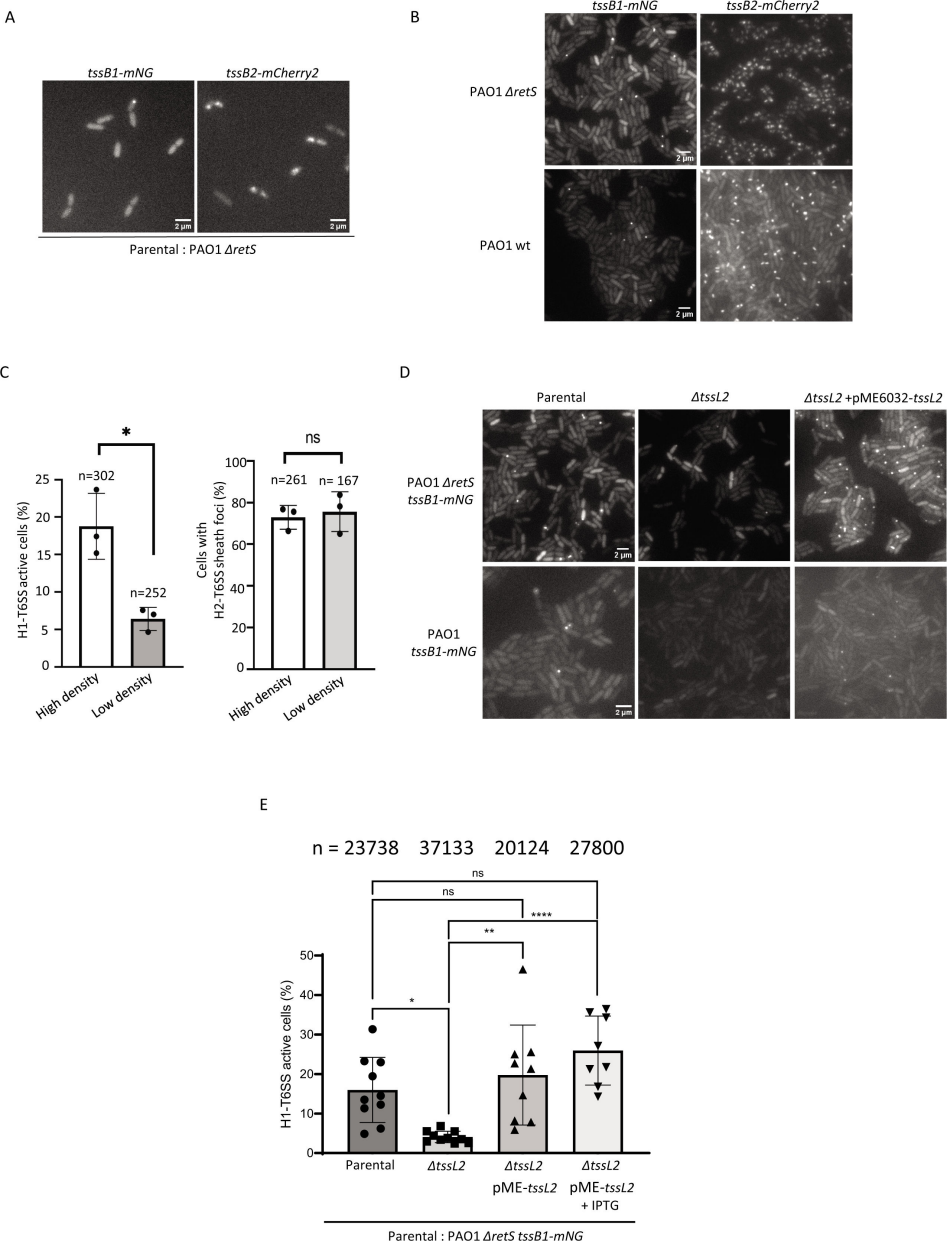
Inactivation of H2-T6SS causes significant reduction in H1-T6SS assembly. Representative images of cells with TssB1-mNG and TssB2-mCherry2 fluorescence signals in (**A**) a low-density field and (B) high-density fields in a wild-type and Δ*retS* genetic background. Scale bar is 2 µm. (**C**) Quantification of cells showing dynamic TssB1-mNG or TssB2-cherry2 foci in high-density and low-density fields. The data are represented as mean ± standard deviation of three independent biological replicates. *P value < 0.1; ns, non-significant; two-tailed Student’s *t*-test. (**D**) Representative images of cells with TssB1-mNG are depicted for each indicated strain. Scale bar is 2 µm. (**E**) Quantification of H1-T6SS activity in Δ*retS* and Δ*retS* Δ*tssL2* mutants, complemented or not with a pME6032-TssL2 plasmid. The strains were spotted on 1% LB-agarose pads and were incubated for 1 h before imaging. The data are represented as mean ± standard deviation of 8–10 fields of view from two independent biological replicates. *P value < 0.05; ordinary one-way ANOVA with multiple comparisons and Tukey post hoc test.

To test the hypothesis that assembly of H2- or H3-T6SS could be triggering H1-T6SS response, we examined the activities of all the three T6SS clusters in a Δ*retS* mutant (21, 36). We constructed chromosomal fusions between genes encoding the T6SS sheath components (*tssB*) and *mNeonGreen* (for the H1-T6SS or the H3-T6SS) or *mCherry2* (for H2-T6SS) encoding genes. We measured single-cell expression levels by flow cytometry in cells incubated under conditions similar to the conditions used below for testing bacterial cell–cell interactions. We detected high expression levels of TssB1-mNG and TssB2-mCh2 in cells that were grown to mid-exponential phase, concentrated and incubated for 3 h on Luria-Bertani (LB) agar. However, no expression of the H3-T6SS could be detected under those conditions (Fig. S1A and B). Furthermore, we performed live-cell fluorescence microscopy imaging of the H1- and H2-T6SS sheath-labeled strains and we observed H1-T6SS sheath dynamics and dueling (defined here as spatially and temporally correlated sheath assembly, contraction, and disassembly in two neighboring cells) as well as multiple dynamic H2-T6SS sheath structures, both in a Δ*retS* and wild-type (WT) background (Fig. 1A and B).

In order to test if the H1-T6SS sheath dynamics (assembly, contraction, disassembly) depend on cell–cell contact, we also measured the TssB1 sheath assembly in cells incubated and imaged at low and high cell density (Fig. 1A and B). We show that about 17% of cells imaged at high density (cells in close contact) assembled H1-T6SS over a period of 3 min, whereas only about 6% of cells assembled H1-T6SS when incubated at low density (minimal cell–cell contact) (Fig. 1C). In contrast, the H2-T6SS sheath assembly was detected in a major subset (around 70%) of the Δ*retS* cells irrespective of cell–cell contact (Fig. 1A through C). Similarly, TssB1-mNG and TssB2-mCh2 assemblies can be identified in a wild-type (*retS* positive) genetic background, albeit with lower overall expression and fewer assembled structures (Fig. 1B). This indicates that *retS* deletion induces H1-T6SS and H2-T6SS expression and assembly, and that the H1-T6SS activity is enhanced by cell–cell contact.

### Inactivation of H2-T6SS reduces H1-T6SS dueling

**Fig 2:**
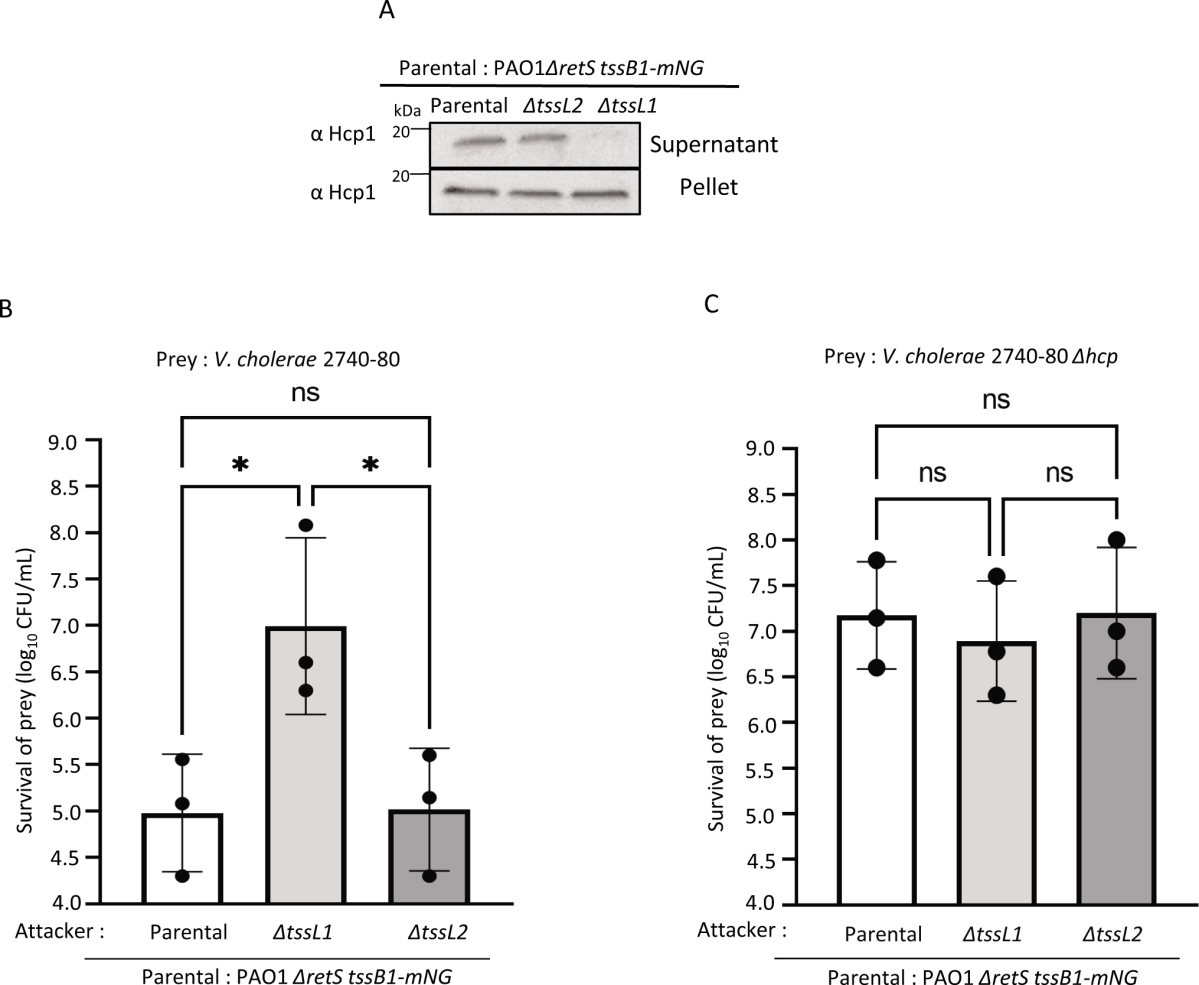
Hcp1 secretion and H1-T6SS retaliation to *V. cholerae* T6SS is independent of H2-T6SS. (**A**) Presence of Hcp1 detected in culture supernatant and cell pellets. The molecular weight is indicated on the left in kilodaltons. (**B**) Summary of competition assays showing recovery of T6SS-positive *V. cholerae* 2740-80 prey strain after co-incubation with different *P. aeruginosa* attacker strains. (**C**) Summary of competition assays showing recovery of T6SS-negative *V. cholerae 2740-80* Δ*hcp* prey strain after co-incubation with different *P. aeruginosa* attacker strains. Data are presented as mean of log_10_ (CFU/mL) of recovered *V. cholerae* 2740-80 strains. Error bars represent standard deviation of three independent replicates. *P value < 0.1; ns, non-significant; ordinary one-way ANOVA with multiple comparisons and Tukey post hoc test.

The observation that cell–cell contact increases H1-T6SS assembly but not H2-T6SS activity under those conditions led us to test the influence of H2-T6SS on H1-T6SS activity. We compared TssB1-mNG assembly in a wild-type or Δ*retS* parental strain and a mutant strain that lacked TssL2, an essential structural component of H2-T6SS (Fig. S1C; Fig. 1D). Remarkably, deletion of *tssL2* significantly reduced the H1-T6SS assemblies both in a wild-type and a Δ*retS* genetic background. In a Δ*retS* strain, we found that 15% of cells display H1-T6SS sheath dynamics compared to only around 5% in the *tssL2*-negative strain. This decrease in H1-T6SS activity was reversed by addition of a copy of *tssL2* in *trans* (Fig. 1E). To test if H2-T6SS-dependent increase in H1-T6SS assembly required cell–cell contact, we also investigated the impact of H2-T6SS on H1-T6SS activity in a liquid culture. Because Hcp secretion is a hallmark of functional T6SS, we monitored the Hcp1 abundance in supernatants harvested from liquid cultures of the parental and *tssL2*-negative strains. Interestingly, the Hcp1 secretion was comparable in the parental and Δ*tssL2* strains (Fig. 2A).

Next, we tested if H2-T6SS had an effect on H1-T6SS ability to counter T6SS attacks from *V. cholerae*. Interbacterial competition experiments showed that the H2-T6SS plays no role in the ability of *P. aeruginosa* to retaliate to *V. cholerae* T6SS-mediated attacks. We recovered about 100-fold less T6SS-positive *V. cholerae* than T6SS-negative *V. cholerae* cells upon incubation with both the parental and *tssL2* deletion *P. aeruginosa* strains (Fig. 2B and C; Fig. S2A and B). This specific inhibition of T6SS-positive *V. cholerae* was dependent on H1-T6SS (Fig. 2B; Fig. S2A and B) as shown previously (31).

Altogether, these observations suggest that H2-T6SS activity can trigger H1-T6SS assembly in a contact-dependent manner. However, the H2-T6SS activity has no influence on H1-T6SS activity in liquid culture or the ability of *P. aeruginosa* H1-T6SS to respond to attacks from other bacteria.

### H1-T6SS responds to H2-T6SS attacks

**Fig 3:**
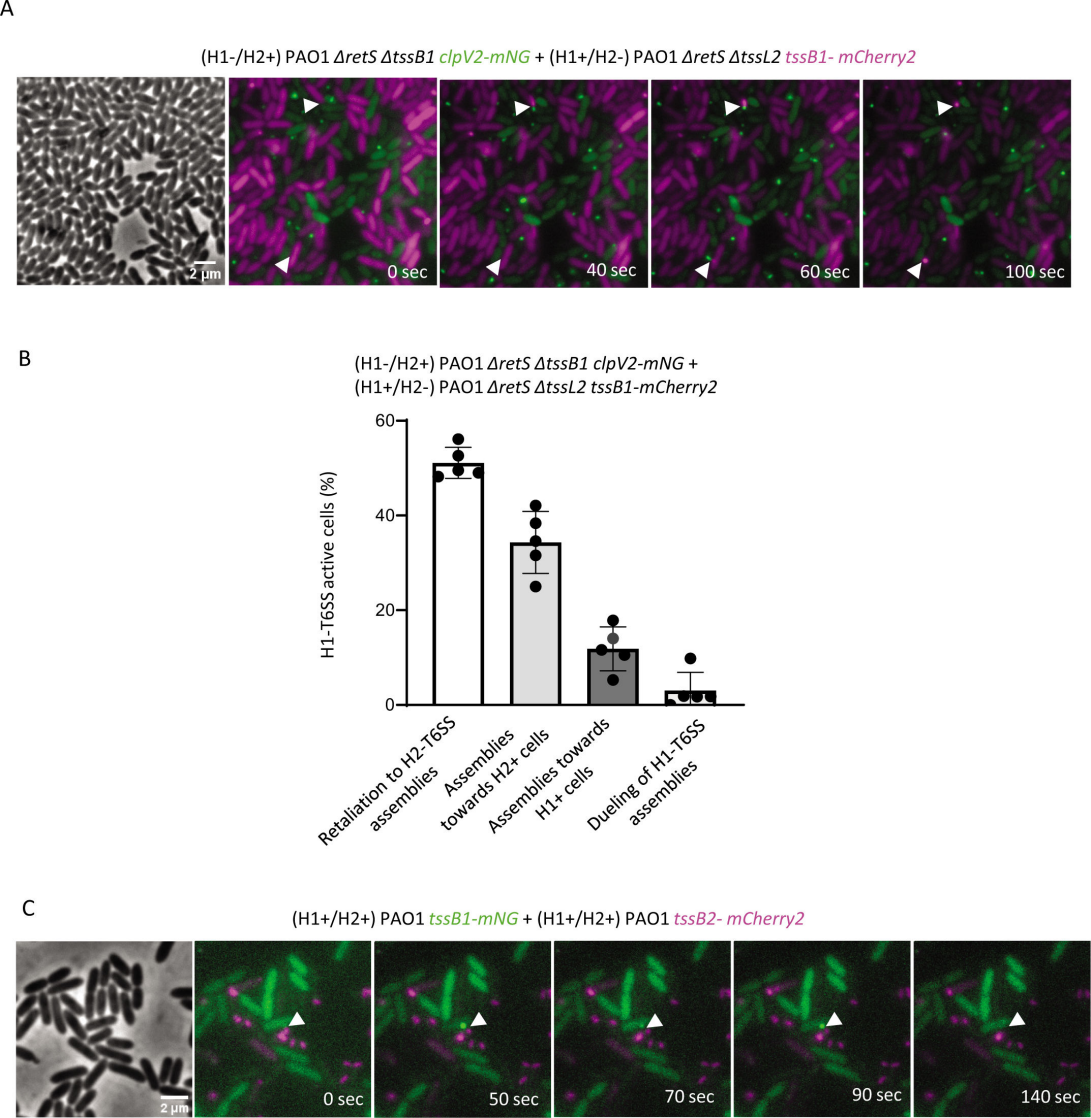
H1-T6SS assembly initiated by H2-T6SS activity. (**A**) H2-T6SS-negative cells (TssB1-mCherry2, magenta) were mixed with H1-T6SS-negative cells (ClpV2-mNG, green), and the H1-T6SS response (TssB1-mCherry2 foci formation) toward H2-T6SS attacks (ClpV2-mNG foci) was monitored. Shown are individual frames of a 5-min time-lapse movie, and the white arrows indicate H1-T6SS response in H2-T6SS-negative cell toward H2-T6SS in H1-T6SS-negative cells. The first frame shows the phase contrast channel, and the next six frames are a merge of GFP and mCherry2 fluorescence channels. The H2-T6SS-negative strain mixed with H1-T6SS-negative mutant at a 1:1 ratio was incubated on 1% LB-agarose pad under a glass coverslip for 1 h before imaging. (**B**) Quantification of H1-T6SS-active H2-T6SS-negative cells in a 1:1 ratio of indicated mixture. H1-T6SS activity was assessed in approximately 30,000 H2-T6SS-negative cells, and from at least 15 100 × 100 µm fields of view. The data are represented as mean ± standard deviation of five independent biological replicates. Number of H1-T6SS-active H2-T6SS-negative cells, *n* = 342. (**C**) Representative images of a retaliation event of TssB1-mNG in a wild-type genetic background against a PAO1 strain carrying a TssB2-mCh2 label in a wild-type genetic background. The event is highlighted with a white arrow in each frame.

The reduction in H1-T6SS assemblies due to inactivation of H2-T6SS prompted us to test if the H1-T6SS can specifically respond to H2-T6SS attacks. To address this issue, we mixed an H1-T6SS-negative strain, Δ*tssB1*, labeled as “H1–/H2+,” and an H2-T6SS-negative strain, Δ*tssL2*, labeled as “H1+/H2–.” We used time-lapse fluorescence microscopy to visualize H1-T6SS assembly by following TssB1-mCherry2 and ClpV2-mNG to detect H2-T6SS activity in a Δ*retS* genetic background. ClpV quickly binds contracted sheaths (21) and therefore its localization can be used to detect T6SS sheath contraction events even in cells with many assembled sheath structures. We show that the ClpV2-mNG foci formation (indicative of H2-T6SS sheath contraction) in H1–/H2+ cells is immediately followed by TssB1-mCherry2 assembly in the neighboring H1+/H2– cells (Fig. 3A). Our analysis shows that about half of the H1-T6SS active cells responded to neighboring H2-T6SS sheath contraction events during 5 min of imaging (Fig. 3B). Moreover, around 35% of H1+/H2– cells fired toward H1–/H2+ cells (Fig. 3C; Fig. S3A and B), suggesting that the observed H1-T6SS assembly in those cells was possibly due to H2-T6SS activity prior to imaging. H1-T6SS assemblies directed toward H1-T6SS-positive cells constituted only about 10% of the total H1-T6SS assembly events, whereas H1-T6SS-triggered events constituted only 1% of the total observed events (Fig. 3B and C; Fig. S3C). Importantly, such retaliation events can also be detected in a wild-type (*retS* positive) genetic background (Fig. 3C).

**Fig 4:**
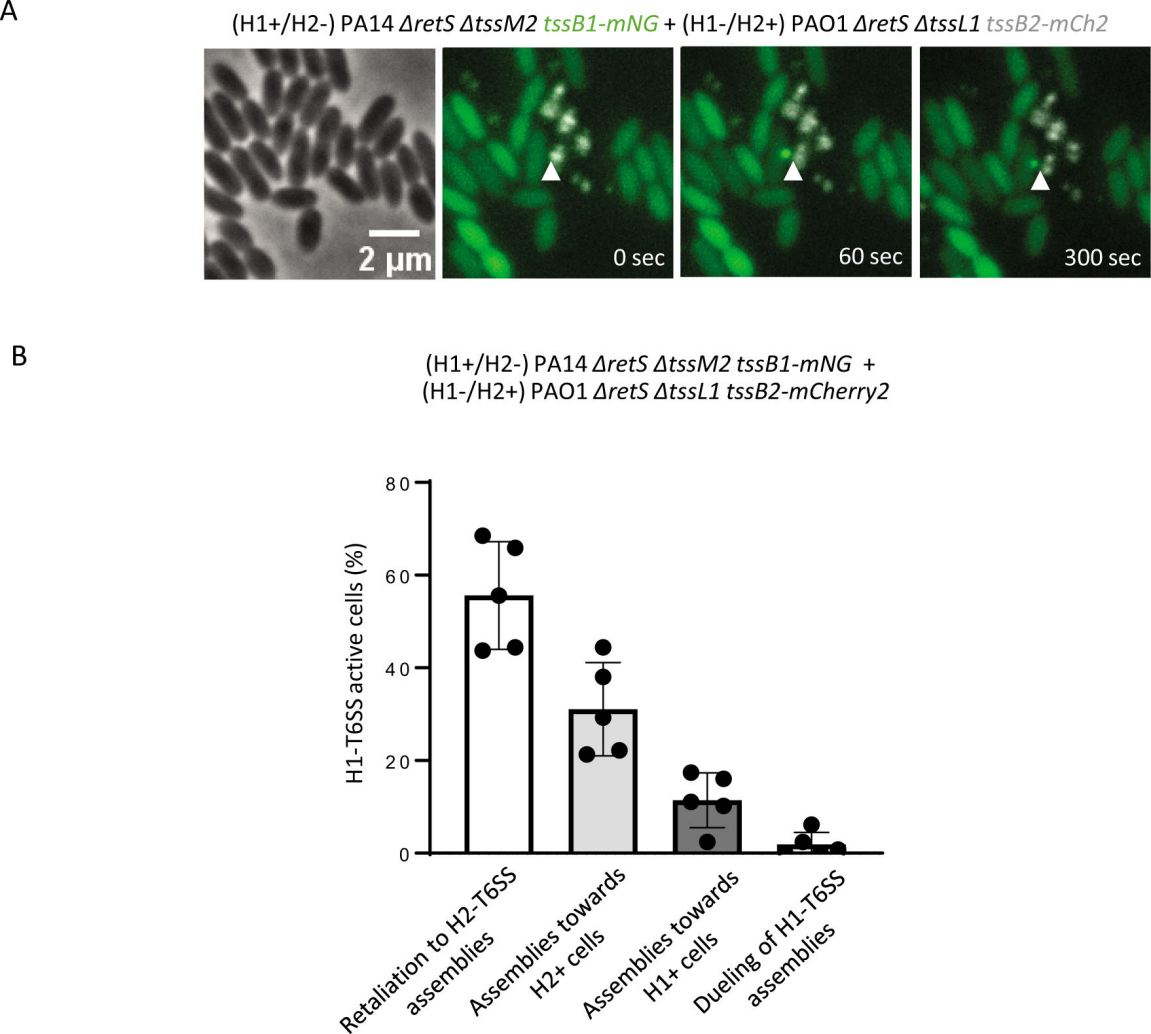
H1-T6SS in PA14 responds to H2-T6SS attacks from PAO1. (**A**) PA14 H2-T6SS-negative cells (TssB1-mNG, green) were mixed with PAO1 H1-T6SS-negative cells (TssB2-mCherry2, grey), and the H1-T6SS response (TssB1-mNG foci formation) toward H2-T6SS attacks (TssB2-mCherry2 foci) was monitored. Shown are individual frames of a 5-min time-lapse movie, and the white arrows indicate H1-T6SS response in H2-T6SS-negative cell toward H2-T6SS in H1-T6SS-negative cells. The first frame shows the phase contrast channel, and the next three frames are a merge of GFP and mCherry2 fluorescence channels. The H2-T6SS-negative strain mixed with H1-T6SS-negative mutant at a 1:1 ratio was incubated on 1% LB-agarose pad under a glass coverslip for 1 h before imaging. (**B**) Quantification of H1-T6SS-active H2-T6SS-negative cells in a 1:1 ratio of indicated mixture. H1-T6SS activity was assessed in approximately 50,000 H2-T6SS-negative cells, and from at least 15 100 × 100 µm fields of view. The data are represented as mean ± standard deviation of five independent biological replicates. Number of H1-T6SS-active H2-T6SS-negative cells, *n* = 495.

To exclude that H1-T6SS assembly is only triggered by H2-T6SS in PAO1 strain, we also performed the same experiments with the PA14 strain. We mixed an H1+/H2– PA14 strain with TssB1-mNG tag and H1–/H2+ PAO1 expressing TssB2-mCherry2 fusion. We show that 56% of H1-T6SS active cells in H1+/H2– PA14 strain were responding to H2-T6SS attack from H1–/H2+ PAO1 (Fig. 4A and B). Nearly 30% of H1-T6SS assemblies in PA14 cells occurred toward H1–/H2+ PAO1 cells (Fig. 4B). The PA14 H1-T6SS dueling represented only about 3% of the total H1-T6SS active events (Fig. 4B).

Together, our results indicate that *P. aeruginosa* retaliates with H1-T6SS in response to incoming H2-T6SS attacks from a neighboring sister cell or cells of another *P. aeruginosa* isolate.

### Loss of H2-T6SS activity confers protection from H1-T6SS

**Fig 5:**
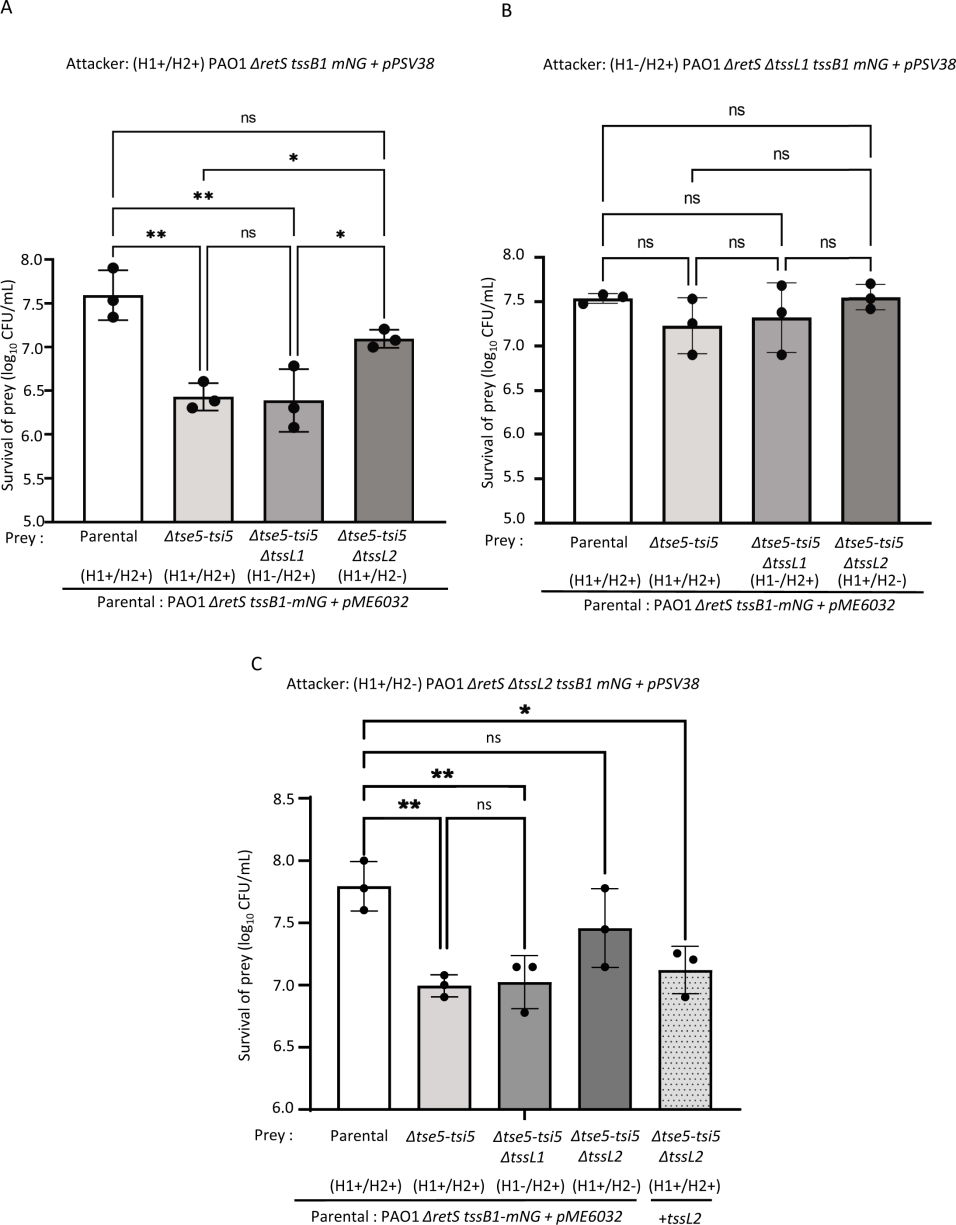
Blocking H2-T6SS in non-immune prey prevents its H1-T6SS-mediated inhibition. Summary of intraspecific competition assays for described *P. aeruginosa* PAO1 attacker and prey strains mixed at a 20:1 ratio. Data depicted as mean of log_10_ (CFU/mL) of recovered prey, post co-incubation with (**A**) Δ*retS tssB1-mNG* attacker, (**B**) Δ*retS* Δ*tssL1 tssB1-mNG* attacker, and (***C***) Δ*retS* Δ*tssL2 tssB1-mNG* attacker. Error bars represent standard deviation of three independent replicates. *P < 0.1, **P < 0.01, ***P < 0.001; ns, non-significant; ordinary one-way ANOVA with multiple comparisons and Tukey post hoc test.

To test if H2-T6SS-triggered H1-T6SS counterattack has any impact on *P. aeruginosa* cell interactions, we tested if the H1-T6SS could kill a non-immune *P. aeruginosa* cell, as a response to H2-T6SS attack. First, we performed in-frame single deletions of H1-T6SS effector-immunity pairs in PAO1 (*tse1-tsi1* to *tse7-tsi7*) and concluded that, under our experimental conditions, most H1-T6SS dependent killing is mediated by Tse5 effector (Fig. S4). Non-immune prey strain lacking *tse5-tsi5* was recovered about 10- to 15-fold less than the parental, immune prey after co-incubation with H1+/H2+ or H1+/H2– attacker strains (Fig. 5A and C), and this inhibition was solely dependent on H1-T6SS activity in the attacker (Fig. 5B). Because *P. aeruginosa* sister cells duel with H1-T6SS (21), we wondered if the elimination of H1-T6SS activity in the prey can minimize its killing. Interestingly, H1-T6SS-negative, non-immune prey was inhibited by H1+/H2+ or H1+/H2– attacker cells similarly to the H1-T6SS-positive non-immune prey (Fig. 5A and C).

To test if inhibition of the non-immune prey is triggered by its H2-T6SS activity, we compared recovery of H1+/H2– and H1+/H2+ non-immune prey cells upon incubation with H1+/H2+ or H1+/H2– attacker cells. Indeed, about three- to five-fold more of the H1+/H2– non-immune prey cells were recovered than the H1+/H2+ non-immune prey cells upon co-incubation with the H1+ attackers (Fig. 5A and Fig. 5C; Fig. S5). Moreover, the expression of *tssL2* in *trans* in H1+/H2– non-immune prey cells restored its inhibition by the H1-T6SS of an H1+/H2– attacker strain (Fig. 5C). These results indicate that H2-T6SS activity in the non-immune prey is responsible for the prey inhibition resulting from the triggered H1-T6SS counterattack.

In addition, we tested if PA14 H1-T6SS attack can be triggered by PAO1 H2-T6SS. PA14 H2-T6SS encodes additional genes, namely, *hcp2*, *vgrG2*, *PA14_43090* encoding a homolog of *Aeromonas hydrophila* T3SS effector, and *PA14_43100* encoding a putative Rhs family protein (37, 38) to which PAO1 has no dedicated immunity proteins. To exclude inhibition by these effectors, we used H2-T6SS-negative PA14 attacker strain for our intraspecific competitions. Importantly, the non-immune PAO1 prey lacking *tse5-tsi5* was recovered almost 10-fold less than the immune prey strain after co-incubation with H2-T6SS-negative PA14 attacker (Fig. S6B through D). This decreased recovery was due to PA14 H1-T6SS attacks as H1-T6SS-negative PA14 caused no inhibition (Fig. S6A). As for the competitions between PAO1 strains, this H1-T6SS-dependent killing was also dependent on the H2-T6SS activity in the prey cells (Fig. S6B). Taken together, this indicates that PAO1 H2-T6SS can trigger counterattack by H1-T6SS in both PA14 and PAO1 strains, and this leads to inhibition of the prey that lacks immunity to H1-T6SS secreted effectors.

### H1-T6SS response is independent of lipase delivery

**Fig 6:**
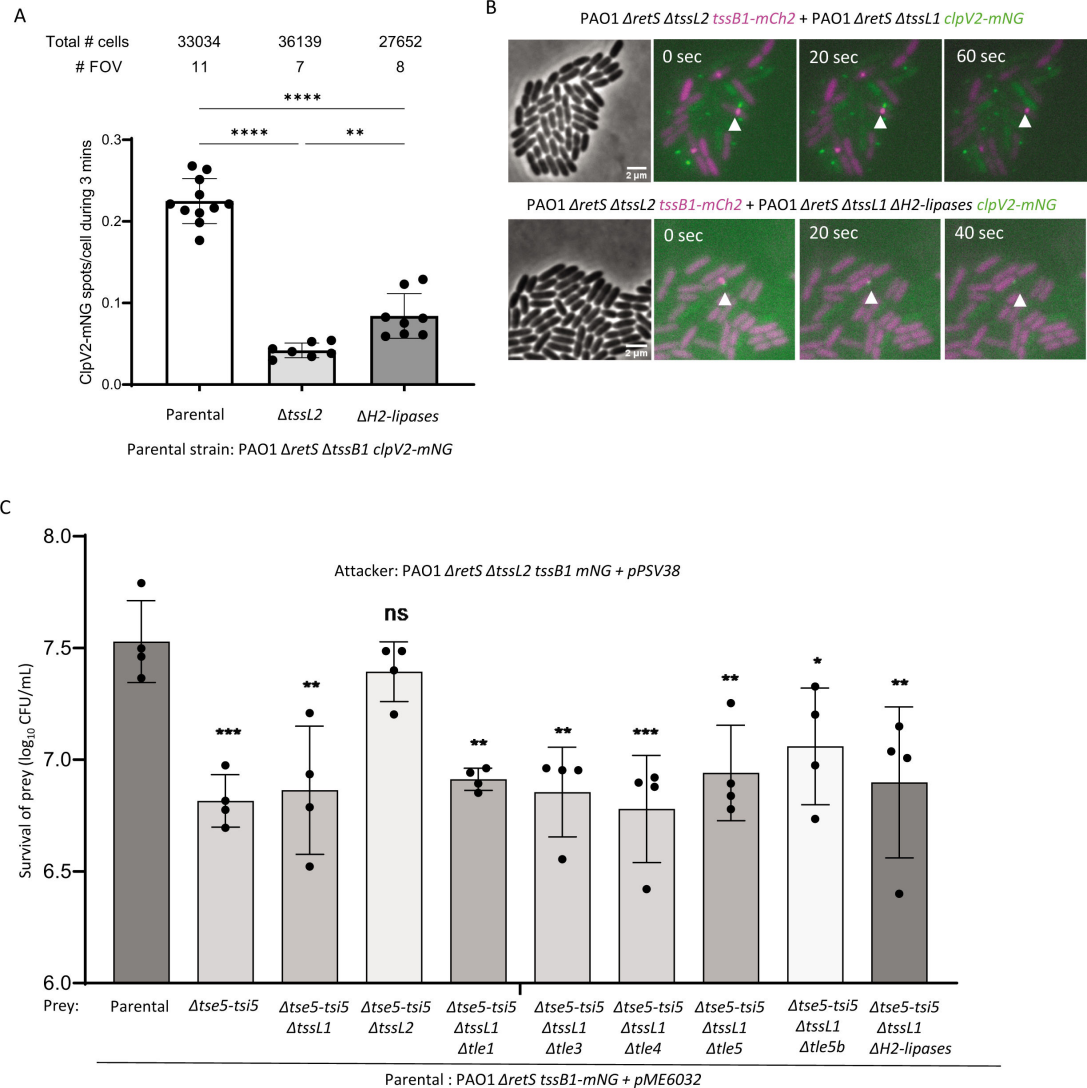
H2-T6SS lipase effectors are dispensable for triggering retaliation events by the H1-T6SS. (**A**) Quantification of the amount of H2-T6SS assemblies/cell during 3 min based on dynamic ClpV2-mNG fluorescent foci. Error bars represent standard deviation of three independent replicates. **P < 0.01, ****P < 0.0001; ordinary one-way ANOVA with multiple comparisons and Tukey post hoc test. (**B**) Microscopy images of H1-T6SS retaliation events induced by a wild-type H2-T6SS or by a mutant lacking all described lipases. (**C**) Survival of *P. aeruginosa* strains lacking the Tse5-Tsi5 effector-immunity pair and different H2-T6SS lipase effectors. Data are presented as mean of log_10_ (CFU/mL). Error bars represent standard deviation of four independent replicates. *P < 0.1, **P < 0.01, ***P < 0.01, ****P < 0.0001; ns, non-significant; ordinary one-way ANOVA with multiple comparisons and Tukey post hoc test.

A previous study suggested that T6SS phospholipase effector TseL from *V. cholerae* V52 is required and sufficient to induce H1-T6SS activity (34). Therefore, we investigated the role of H2-T6SS lipase effectors, namely, Tle1, Tle3, Tle4/TplE, Tle5a/PldA, and Tle5b/PldB (9, 39–42), in eliciting an H1-T6SS response. We deleted all these lipase effectors (referred to as Δ*H2-lipases* strain) and tested the H2-T6SS activity of the resulting mutant strain by quantifying ClpV2-mNG dynamics by live-cell imaging. The Δ*H2-lipases* strain had H2-T6SS activity reduced to 30% of the parental strain (Fig. 6A). This reduction could be due to partial defects in T6SS assembly caused by removal of multiple T6SS effectors as previously described (43, 44).

Next, we assessed the ability of the Δ*H2-lipases* strain to elicit H1-T6SS retaliation by both microscopy and intraspecific competition. Remarkably, we observed that the Δ*H2-lipases* strain induced H1-T6SS retaliation events (Fig. 6B). Moreover, the Δ*H2-lipases* strain, as well as all single H2-T6SS lipase effector deletion mutants that lacked the Tse5-Tsi5 effector-immunity pair, was killed in an H1-T6SS-dependent manner by an attacker strain lacking an active H2-T6SS (Fig. 6C; Fig. S7A).

**Fig 7:**
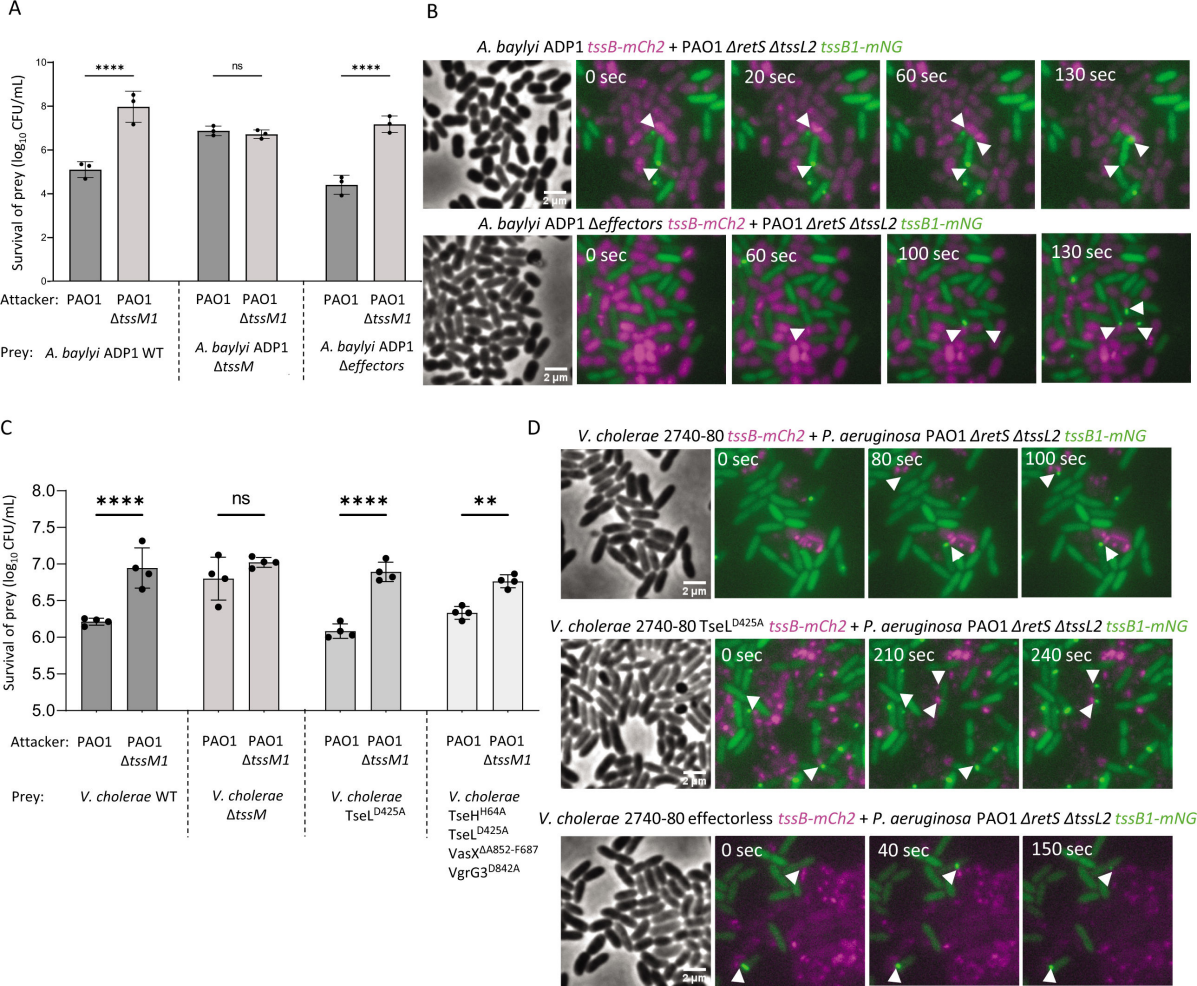
The T6SS of *A. baylyi* and *V. cholerae* induces H1-T6SS retaliation independently of known effectors. (**A**) Survival of *A. baylyi* ADP1 strains during competition with H1-T6SS-positive or negative *P. aeruginosa*. Error bars represent standard deviation of three independent replicates. ****P value < 0.0001; ns, non-significant; ordinary one-way ANOVA with multiple comparisons and Tukey post hoc test. Data are presented as mean of log_10_ (CFU/mL). (**B**) Microscopy images showing retaliation events triggered by WT or T6SS-effector-negative *A. baylyi*. (**C**) Survival of *V. cholerae* 2740-80 strains incubated with H1-T6SS-positive or negative *P. aeruginosa*. Error bars represent standard deviation of three independent replicates. **P value < 0.01, ****P value < 0.0001; ns, non-significant; ordinary one-way ANOVA with multiple comparisons and Tukey post hoc test. Data are presented as mean of log_10_ (CFU/mL). (**D**) Microscopy images showing retaliation events triggered by WT or T6SS-effector inactivated *V. cholerae*.

Finally, we also tested whether the T6SS of other organisms lacking lipase effectors could trigger H1-T6SS responses. As previously reported (35), an *A. baylyi* ADP1 mutant lacking five known effectors (including the putative lipase effector) is killed by *P. aeruginosa* in an H1-T6SS-dependent manner (Fig. 7A; Fig. S7B). In addition, live-cell imaging shows that H1-T6SS retaliation occurs in response to T6SS activity of *A. baylyi* ADP1 effector-negative mutant (Fig. 7B). Furthermore, a *V. cholerae* mutant of strain 2740-80 carrying an inactive version of TseL, as well as a strain carrying all four inactivated antibacterial effectors, TseL^D425A^, VgrG3^D842A^, TseH^H64A^, and VasX^ΔA852-F867^ (mutations reported previously [44]), was killed by H1-T6SS (Fig. 7C; Fig. S7C). In addition, live-cell imaging showed that these T6SS effector-inactivated *V. cholerae* 2740-80 mutants triggered an H1-T6SS response (Fig. 7D). Taken together, our results indicate that T6SS effectors with lipase activity are dispensable for eliciting an H1-T6SS response.

## DISCUSSION

In this study, we aimed to identify the mechanism that triggers the spontaneous H1-T6SS assemblies in *P. aeruginosa* cells. Previous reports showed that treatment with polymyxin B, extracellular DNA, and EDTA chelator, as well as T4SS and T6SS attacks from sister cells or other heterologous bacteria, can activate H1-T6SS via the TagQRST signaling pathway (21, 31–33). Interestingly, sensing of kin cell lysis by the Gac/Rsm pathway was shown to lead to an elevated expression of genes that encode H1-T6SS components (45). However, spontaneous H1-T6SS assemblies that arise in *P. aeruginosa* Δ*retS* as well as in RetS-positive cells, termed dueling, were poorly understood. The fact that the H1-T6SS can sense external T6SS attacks motivated us to examine if H2-T6SS/H3-T6SS activity of a sister cell could trigger H1-T6SS response. However, the expression of only H1 and H2-T6SS was detected in *P. aeruginosa* PAO1 Δ*retS* by flow cytometry (Fig. S1A and B).

Our microscopic analysis of *P. aeruginosa* cells on a solid agarose pad indicated that absence of H2-T6SS led to a significant decrease in H1-T6SS assemblies (Fig. 1D and E). On the other hand, the ability to retaliate to T6SS-mediated attacks by *V. cholerae* was independent of H2-T6SS (Fig. 2B). Because Hcp1 was also present in the culture supernatant of H2-T6SS-negative mutant (Fig. 2A), we concluded that the decreased assembly of the H1-T6SS in an H2-T6SS-negative strain is not due to a structural defect in the assembly of the H1-T6SS. We hypothesized that H1-T6SS activity is triggered by H2-T6SS only when sister cells are in close contact on a solid surface. Indeed, time-lapse imaging experiments revealed that H1-T6SS is assembled in direct response to H2-T6SS attacks from a neighboring cell, or toward cells that had an active H2-T6SS (Fig. 3A through C). Importantly, H1-T6SS in PA14 also responded to H2-T6SS attack from PAO1 (Fig. 4A and B), which indicated that H1-T6SS retaliation to H2-T6SS attacks happens in between *P. aeruginosa* strains.

Competition experiments showed that a lethal H1-T6SS counterattack from an attacker strain is dependent on H2-T6SS activity in the non-immune prey. The non-immune prey without a functional H2-T6SS was spared from H1-T6SS-mediated inhibition (about three- or five-fold increase in its recovery) (Fig. 5A and C). Importantly, the observed H2-T6SS-mediated triggering of H1-T6SS can also be observed in strain PA14 (Fig. S6B).

**Fig 8:**
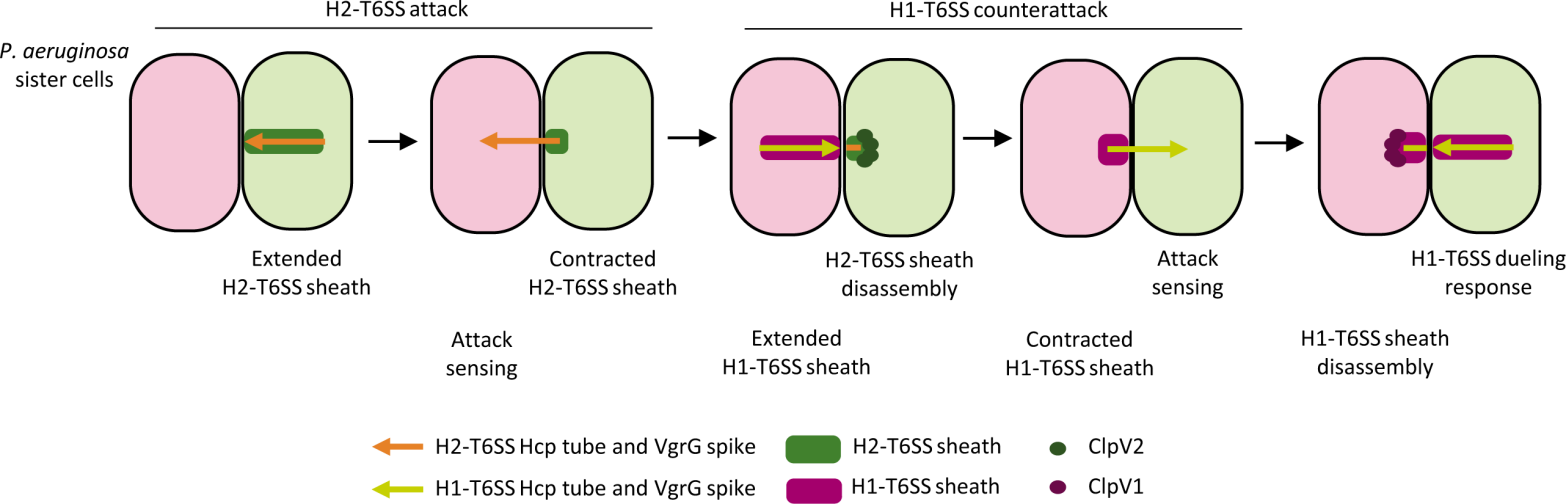
Model of H2-T6SS-mediated triggering of H1-T6SS activity and dueling in *P. aeruginosa*. The H2-T6SS sheath contraction in initiator *P. aeruginosa* cell (green shade) powers the delivery of H2-Hcp and H2-VgrG spike into neighboring sister cell (magenta shade). Consequently, the H1-T6SS is triggered in the neighboring cell, which delivers the H1-Hcp and H1-VgrG into the initiator cell. Finally, the H1-T6SS dueling response is triggered in the initiator cell. The sheath contraction events coincide with the respective ClpV–sheath interactions.

Altogether, we propose a model that describes the events that lead to H1-T6SS dueling between sister cells (Fig. 8). H2-T6SS attack from an initiator cell (Fig. 8, green shade) can elicit an H1-T6SS response from the neighboring cell (Fig. 8, magenta shade). As a retaliation to this response, the H1-T6SS is assembled precisely toward the initiator. Hitting the initiator cell with H1-T6SS will trigger localized assembly of its H1-T6SS and thus result in rounds of H1-T6SS assemblies in those two cells, observed as dueling (Fig. 8). However, the exact mechanism on how H2-T6SS activates the TagQRST sensory module remains unknown. An earlier study described the role of *V. cholerae* V52 T6SS effector TseL in activating the TagQRST cascade (34). However, our results show that *V. cholerae* 2740-80 T6SS without any effector activity can effectively trigger an H1-T6SS response (Fig. 7C and D). We also found that H2-T6SS phospholipase effectors Tle1, Tle3, Tle4/TplE, Tle5a/PldA, and Tle5b/PldB, although essential for fully active H2-T6SS, are not required to elicit an H1-T6SS response (Fig. 6B and C). Finally, an *A. baylyi* mutant strain lacking all five known T6SS effectors, including a lipase, was shown to elicit H1-T6SS responses as previously described (35). Taken together, our study supports the model that H1-T6SS can be activated independently of effector activity.

Our findings shed light on *P. aeruginosa* intraspecific competitions that may occur in various habitats. It was shown earlier that *P. aeruginosa* strains, PAO1, and PAK had differences in H1-T6SS toxin-immunity pair *tse7-tsi7*. Intriguingly, the Tsi7^PAK^ was unable to block the toxic effects of Tse7^PAO1^ and therefore PAO1 could outcompete PAK (46). Furthermore, the three T6SS clusters were reported to be upregulated in *P. aeruginosa* PAO1 biofilm cells (47), which suggests that the H1-T6SS might also respond to H3-T6SS puncture in conditions that are conducive to H3-T6SS assembly. *P. aeruginosa* biofilm is regulated by effective cell–cell communication mediated by quorum sensing (5, 48), and H1-T6SS response to H2-T6SS may affect intercellular signaling in the course of biofilm formation. Our results also suggest that the interplay between the different T6SS clusters in *P. aeruginosa* can influence the outcome of interstrain competitions, particularly when effector-immunity pairs differ between strains, as is often the case in clinical isolates of *P. aeruginosa* (49), and could promote colonization by specific isolates in lung microenvironments of CF patients. Considering the role of *P. aeruginosa* biofilms in long-term persistence, infection of CF patients, and antimicrobial resistance, additional research is needed to understand T6SS-mediated cell interactions within biofilms and during infection (5, 10, 50, 51).

## MATERIALS AND METHODS

### Bacterial strains and growth conditions

*P. aeruginosa* PAO1/PA14 ∆*retS*-labeled strains, as well as knockout strains, were generated as described previously (31). A list of strains used can be found in Table S1. Recombinant clones were checked by colony PCR and were sequence verified. A list of plasmids used in this study can be found in Table S2. Bacteria were grown in LB broth at 37°C. Liquid cultures were grown aerobically. *E. coli* DH5α was used as a cloning strain; genetic manipulations in *P. aeruginosa* were carried out using *E. coli* SM10 λpir for conjugation. Antibiotics used were streptomycin (100 µg/mL for *V. cholerae* and *A. baylyi*), tetracycline (100 µg/mL for *P. aeruginosa* and 12.5 µg/mL for *E. coli*), and gentamicin (20 µg/mL).

### Flow cytometry

Bacteria with different fluorescently labeled T6SS sheaths (TssB1-mNG, TssB2-mCh2, or TssB3-mNG) were grown to mid-exponential phase at 37°C from an overnight culture in LB and measured, or concentrated to an optical density (OD) of 10, spotted on an LB agar plate and incubated for 3 h at 37°C, then resuspended and measured. Prior to measurement, all samples were diluted 100× in phosphate-buffered saline (PBS) and were analyzed by a BD Fortessa flow cytometer, with 950 V green and 950 V red laser on a high-throughput system (HTS) in a high-throughput mode, with a sample flow rate of 1 µL/s for a total of 10 µL of sample. Data were analyzed using FlowJo v10, and the median fluorescence intensity (MFI) was plotted using Prism.

### Bacterial killing assays

Quantitative killing assays were performed as described previously (31, 52). Briefly, overnight cultures were diluted 1 to 100 into fresh LB. Bacterial pellets were harvested at OD ≈ 1, washed twice in 1 mL LB and concentrated 10 times (OD ≈ 10). Indicated strains were mixed at a ratio of 10:1 (*P. aeruginosa* to *V. cholerae*), and 5 µL of the mixtures was spotted on dry LB plates and incubated for 3 h at 37°C. Subsequently, bacterial spots were cut out, and cells were resuspended in 0.5 mL LB. Cell mixtures were spotted in serial dilutions (1:10) on selective recovery plates (streptomycin for *V. cholerae*, irgasan for *P. aeruginosa*). CFU was counted after ≈16 h of incubation at 37°C. Three independent biological replicates were analyzed.

### Intraspecific competition assays

For intraspecific competition experiments using *P. aeruginosa* strains, plasmids with antibiotic resistance markers for prey and attacker strains were used. In prey strains, we electroporated pME6032 vector (53) (that encodes the gene for tetracycline resistance), and attacker strain was transformed with pPSV38 vector (54) (gentamicin resistance marker), by conjugation. Cultures were treated in the same manner as described in bacterial killing assays, but with some variations. Instead of concentrating the cultures to OD ≈ 10, we normalized to OD ≈ 1. Furthermore, the attacker and prey strains were mixed at a 20:1 ratio. Then, 5 µL of cell mixture was spotted on LB agar plates and incubated for 16 h at room temperature. Afterward, the bacterial spots were excised, resuspended in LB, and spotted in serial dilutions as described in bacterial killing assays. The attacker strains were selected on LB plates with gentamicin antibiotic, and prey strains were selected on LB plates with tetracycline. Three or four independent biological replicates were analyzed.

### Electroporation in *P. aeruginosa*

Electroporation to deliver plasmid into *P. aeruginosa* was performed similarly as described earlier (55). Briefly, 2 mL overnight grown culture was spun down in a tabletop microcentrifuge for 1 min at room temperature. The supernatant was discarded, and the pellet was washed twice with 800 µL of ddH_2_O. The final pellet was dissolved in an appropriate volume of ddH_2_O, typically 100 to 500 µL. One-microliter amounts of plasmid were added to 100 µL of cells in 2-mm cuvettes, and electroporation was performed using a Bio-Rad GenePulser with the following settings: 25 µF, 400 Ω, 1.8 kV. All steps described were performed at room temperature. LB broth (1 mL) was added immediately after the pulse, and cells were incubated for 2 h at 37°C with shaking before plating on selective plates.

### Western blots

Experiments were performed as described previously (56). Bacteria were cultivated as described for the bacterial killing assay. Proteins in 900 µL culture supernatant were precipitated by TCA/acetone. Primary antibodies were used at a final concentration of 1 µg/mL in 5% milk in Tris-buffered saline (pH 7.4) containing 0.1% Tween (TBST). Secondary antibodies were incubated for 1 h with horseradish peroxidase-labeled anti-rabbit antibody (Jackson Lab), washed with TBST, and peroxidase was detected by LumiGLO Chemiluminescent Substrate (Cell Signaling Technology, USA) on a gel imager (GE ImageQuant LAS 4000). To monitor protein abundance in cell pellets, 100 µL of culture was centrifuged, and the supernatant was discarded. For detection of Hcp1 in cell pellets, 100 µL cells was harvested, resuspended in 100 µL Laemmli buffer, and boiled for 10 min at 95°C. Proteins were separated on Novex 4%–12% Bis-Tris SDS–polyacrylamide gel electrophoresis gels (Thermo Fisher Scientific) and were transferred to nitrocellulose membrane for immuno-detection.

### Fluorescence microscopy

Procedures similar to those described previously (21, 31, 36) were used to detect fluorescence signal in *P. aeruginosa*. Overnight cultures of *P. aeruginosa* were washed in LB, diluted 1:100 into fresh medium, and cultivated for 2.5–3.0 h to an OD at 600 nm of about 0.8–1.2. Cells from 1 mL of the culture were resuspended in 50–100 µL of fresh LB, concentrated to OD 10 to achieve high cell density. For low cell density imaging without cell–cell contact, cell suspension with OD ~ 1 was used. Cell suspensions were placed on a thin pad of 1% agarose in LB and were covered with a glass coverslip. Furthermore, the cells under coverslip were incubated at 37°C for 1 h and then imaged. Cells close to the periphery of the agarose pad were imaged. Nikon Ti-E inverted motorized microscope with Perfect Focus System and Plan Apo 100× Oil Ph3 DM (NA 1.4) objective lens were used. Spectra X light engine (Lumencor), ET-GFP (Chroma 49002), and ET-mCherry (Chroma 49008) filter sets were used to excite and filter fluorescence. The microscope was equipped with SPECTRA X light engine (Lumencor), and ET-EGFP (Chroma #49002) and ET-mCherry (Chroma #49008) filter sets were used to excite and filter fluorescence. The setup further contained a sCMOS camera pco.edge 4.2 (PCO, Germany) (pixel size 65 nm) and VisiView software (Visitron Systems, Germany) to record images. Temperature control was set at 30°C, and humidity was adjusted to 95% by an Okolab T-unit (Okolab). For time-lapse imaging of bacterial mixtures (Fig. 3A; Fig. S3A and S5A), the cultures were prepared and concentrated to OD 10 (as described above) and mixed at a 1:1 ratio. Then, 2 µL of this mixture was spotted on a thin pad of 1% agarose in LB, covered with a coverslip and incubated at 37°C for 1 h before imaging.

### Image analysis

Fiji (57) was used for all image analysis and manipulations as described previously (31). For quantification of T6SS activity, total cell number was assessed using the “find maxima” options with noise tolerance adjusted manually for each case. All quantifications were verified manually and carried out using the “edge maxima exclusion” function. For quantification of T6SS activity from time-lapse movies, the “temporal color code” function was used. For high-density conditions, three different 15 × 15 µm fields were used; for low-density conditions, at least 10 different 50 × 50 µm fields were used for counting. The total number of cells analyzed (*n*) is indicated in the graphs (Fig. 1C). For quantification of H1-T6SS activity in Δ*retS* and Δ*retS* Δ*tssL2* mutants, five different 130 × 130 µm fields were used, and the total number of analyzed cells is indicated in Figure 1E. The total number of cells was estimated using the Find Maxima function with a prominence of 3000 on the phase contrast channel, and the amount of TssB1-mNG assemblies was estimated with the Find Maxima function with a prominence of 700 on the GFP channel. For quantification of H1-T6SS response to H2-T6SS and spontaneous H1-T6SS activity in mixtures, “cell counter” plugin was used. Quantification of ClpV2-mNG foci in monoculture (Fig. 6A) was performed with TrackMate v7.11.1 (58) using the DoG detector mode with an estimated blob diameter of 0.25 µm and a threshold of 20, using median filtering and subpixel localization. Tracks were calculated with the Simple LAP tracker tool with a linking maximum distance of 0.2 µm, a gap-closing maximum distance of 0.4 µm, and a gap-closing maximum frame of 1. Tracks with a maximum duration of more than 12 frames (2 min) were excluded. The remaining tracks in each field of view were normalized by the amount of cells in the field, as estimated by the Find Maxima function with a light background (phase contrast channel) with a prominence of 3000. All imaging experiments were performed with at least three to five biological replicates.

### Statistics

Ordinary one-way analysis of variance (ANOVA) with multiple comparisons and Tukey post hoc test was used to determine the significance between all groups using GraphPad Prism version 9.3.1. Two-tailed Student’s *t*-test was performed when comparing two means. If not indicated differently, data are represented as mean ± standard deviation (SD).

## SUPPLEMENTAL MATERIAL

Supplemental material mbio.00355-24-s0001.pdf Supplemental figures and tables.

ASM does not own the copyrights to Supplemental Material that may be linked to, or accessed through, an article. The authors have granted ASM a non-exclusive, world-wide license to publish the Supplemental Material files. Please contact the corresponding author directly for reuse.
