# Supplementary material for: Initiation of H1-T6SS dueling between Pseudomonas aeruginosa
Source: mBio. 2024 Jul 11;15(8):e00355-24. doi: 10.1128/mbio.00355-24 (PMC11323562; doi:10.1128/mbio.00355-24)
Supplement: Supplemental material — Supplemental figures and tables. [file mbio.00355-24-s0001.pdf]

Supplementary Information

Initiation of H1-T6SS dueling between *Pseudomonas aeruginosa*

M. George*, S. Narayanan*, A. Tejada-Arranz*, A. Plack, M. Basler

Biozentrum, University of Basel, Spitalstrasse 41, CH-4056 Basel, Switzerland

* contributed equally

Supplementary Figure 1

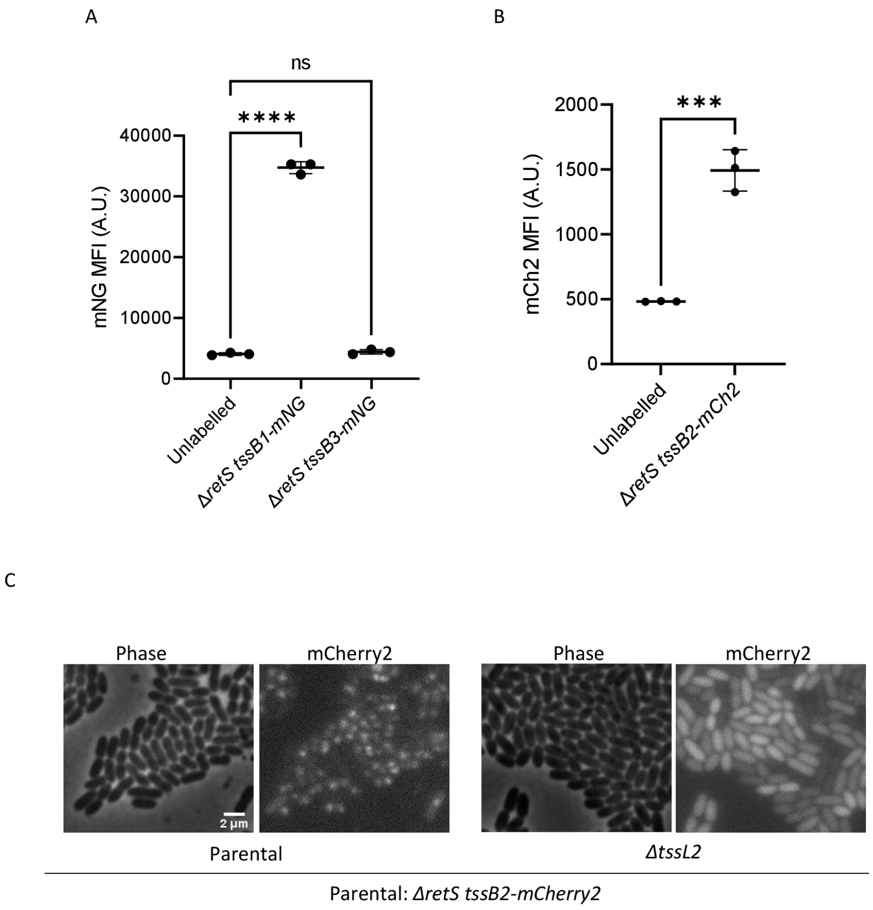

Supplementary Figure 1. The expression of the H1- and H2-T6SSs.

(A) Median fluorescence intensity (MFI) of mNeonGreen (mNG) when fused to TssB1 or TssB3 in a Δ*retS* genetic background. Error bars represent standard deviation of three independent replicates. ****p-val<0.0001, ns- non-significant; Ordinary one-way ANOVA with multiple comparisons and Tuckey post hoc test. (B) MFI of mCherry2 (mCh2) when fused to TssB2 in a Δ*retS* genetic background. Error bars represent standard deviation of three independent replicates. ***p-val<0.001, two –tailed Student’s t test. (C) Fluorescence microscopy images of parental and Δ*tssL2* TssB2-mCherry2 labeled strains.

Supplementary Figure 2

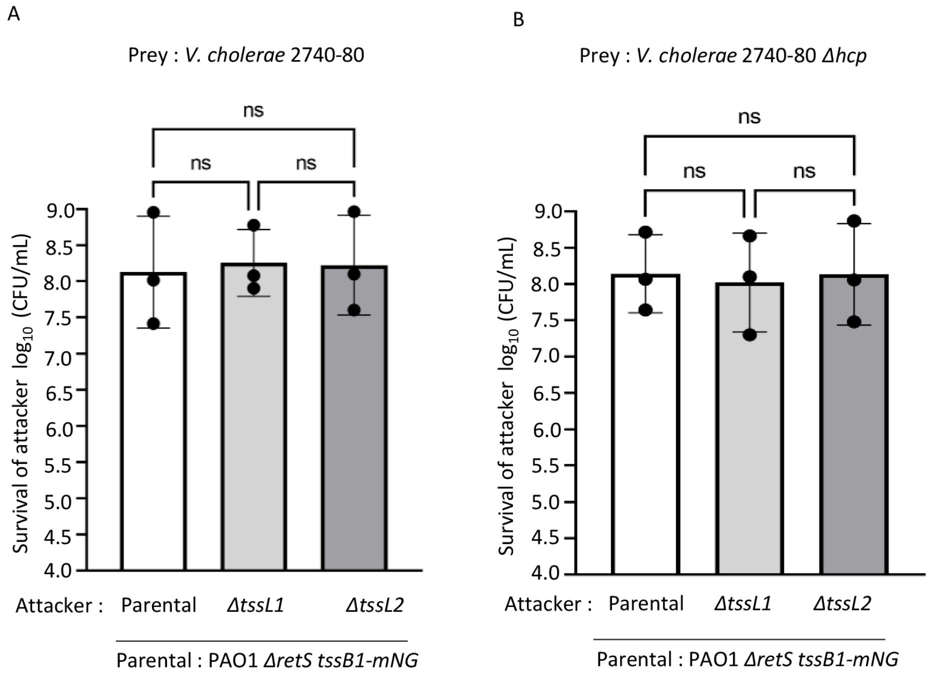

Supplementary Figure 2. Recovery of *P. aeruginosa* upon competition with *V. cholerae*.

(A) Summary of competition assays showing recovery of different *P. aeruginosa* strains (in Figure 2B) after co-incubation with T6SS positive *V. cholerae 2740-80 prey* strain. (B) Summary of competition assays showing recovery of different *P. aeruginosa* strains (in Figure 2C) after co-incubation with T6SS negative *V. cholerae 2740-80 Δhcp* prey strain. Data are presented as mean of Log_{10}(CFU/mL) of recovered *P. aeruginosa* strains. Error bars represent standard deviation of three independent replicates. ns- non-significant; Ordinary one-way ANOVA with multiple comparisons and Tuckey post hoc test.

Supplementary Figure 3

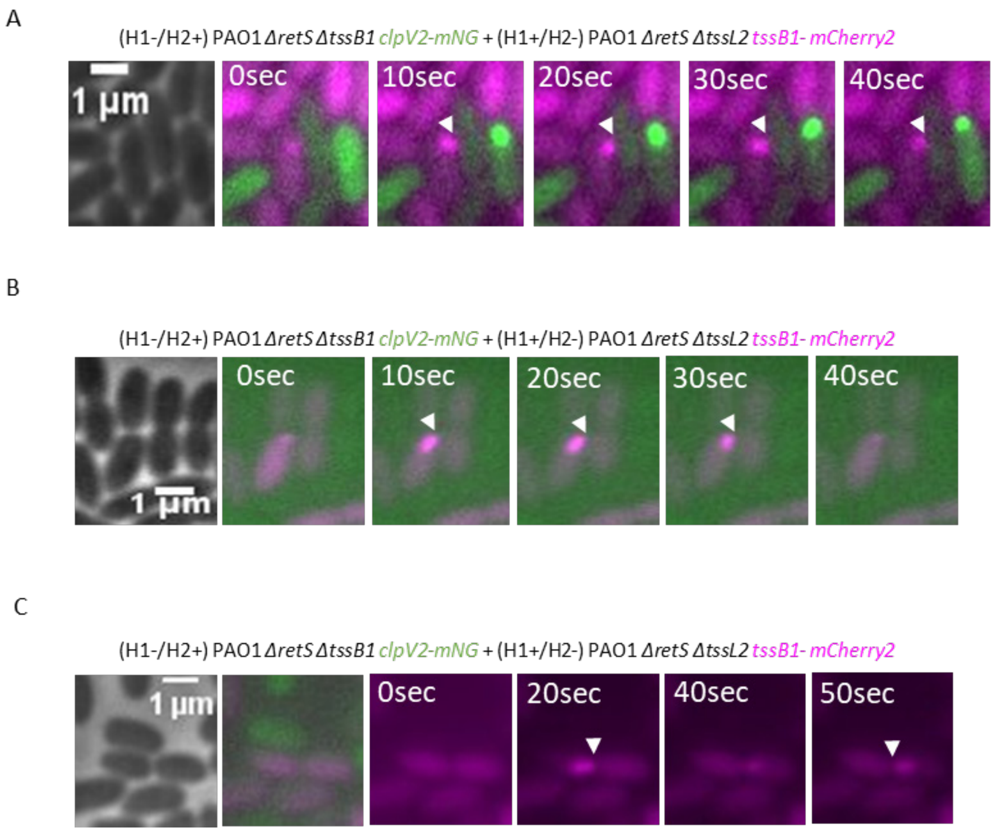

Supplementary Figure 3. Spontaneous H1-T6SS activity in cells next to H1-/H2+ or H1+/H2- cells.

(A) Individual frames of a 5 min time-lapse movie and white arrow indicate H1-T6SS spontaneous assembly negative H1+/H2- cell towards a H1-/H2+ cell. The first frame shows the phase contrast channel and the next five frames are a merge of GFP and mCherry2 fluorescence channels. (B) Individual frames of a 5 min time-lapse movie and white arrow indicate an H1-T6SS spontaneous assembly in H1+/H2- cell towards a H1+/H2- cell. The first frame shows the phase contrast channel and the next five frames are a merge of GFP and mCherry2 fluorescence channels. (C) Individual frames of a 5 min time-lapse movie and white arrow indicate H1-T6SS dueling response in an H1+/H2- cell that is in contact with another H1+/H2- cell. The first frame shows the phase contrast channel. The second frame is a merge of GFP and mCherry2 fluorescence channels. The subsequent four frames show mCherry2 fluorescence channel.

Supplementary Figure 4

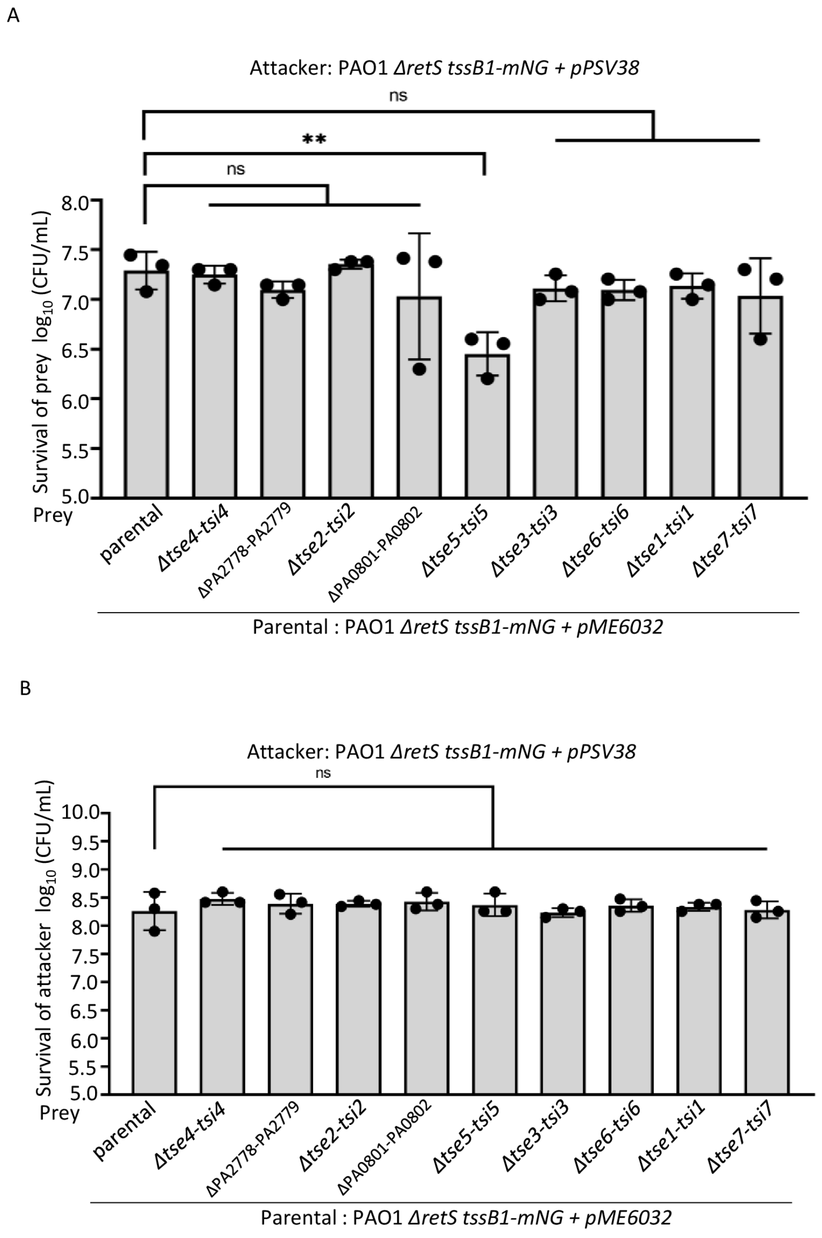

Supplementary Figure 4. Tse5 mediated killing of *P. aeruginosa* PAO1.

(A) Summary of intraspecific competition assays for described *P. aeruginosa* PAO1 attacker and prey strains mixed at 20:1 ratio. Data depicted as mean of Log_{10}(CFU/mL) of recovered prey strains after co-incubation with *ΔretS* attacker. (B) Data depicted as mean of Log_{10}(CFU/mL) of recovered attacker after co-incubation with prey strains (in Supplementary Figure 4A). Error bars represent standard deviation of three independent replicates. **p-val<0.01, ns- non-significant; Ordinary one-way ANOVA with multiple comparisons and Tuckey post hoc test.

Supplementary Figure 5

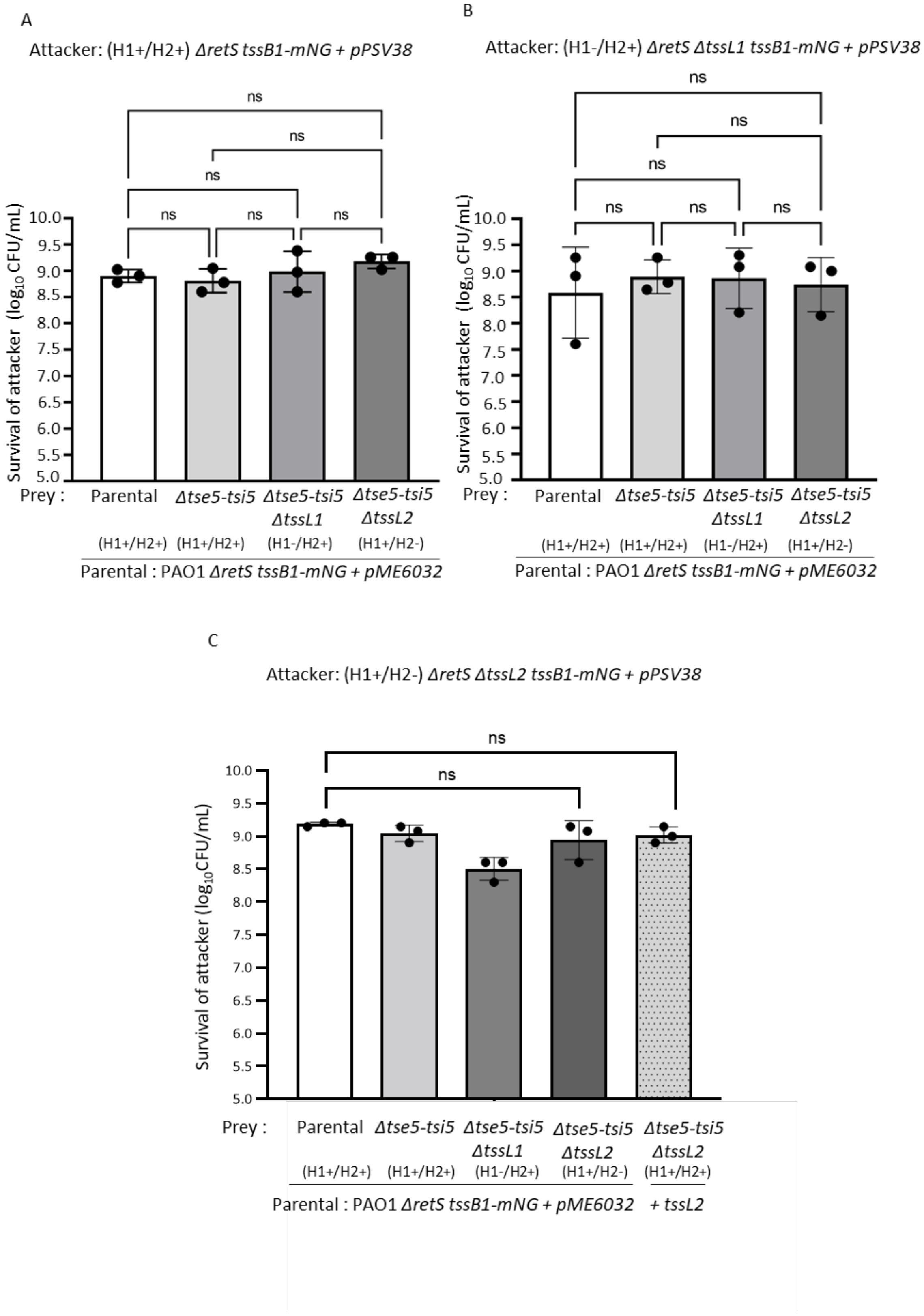

Supplementary Figure 5. Protection of non-immune prey from H1-T6SS.

Summary of intraspecific competition assays for described *P. aeruginosa* PAO1 attacker and prey strains mixed at 20:1 ratio. Data depicted as mean of Log_{10} (CFU/mL) of recovered (A) *ΔretS tssB1-mNG*

attacker, after co-incubation with different prey strains (used in Figure 5A), (B) *ΔretS ΔtssL1 tssB1-mNG* attacker, after co-incubation with different prey strains (used in Figure54B) and (C) *ΔretS ΔtssL2 tssB1-mNG* attacker, after co-incubation with different prey strains (used in Figure 5C). Error bars represent standard deviation of three independent replicates. ns- non-significant; Ordinary one-way ANOVA with multiple comparisons and Tuckey post hoc test.

Supplementary Figure 6

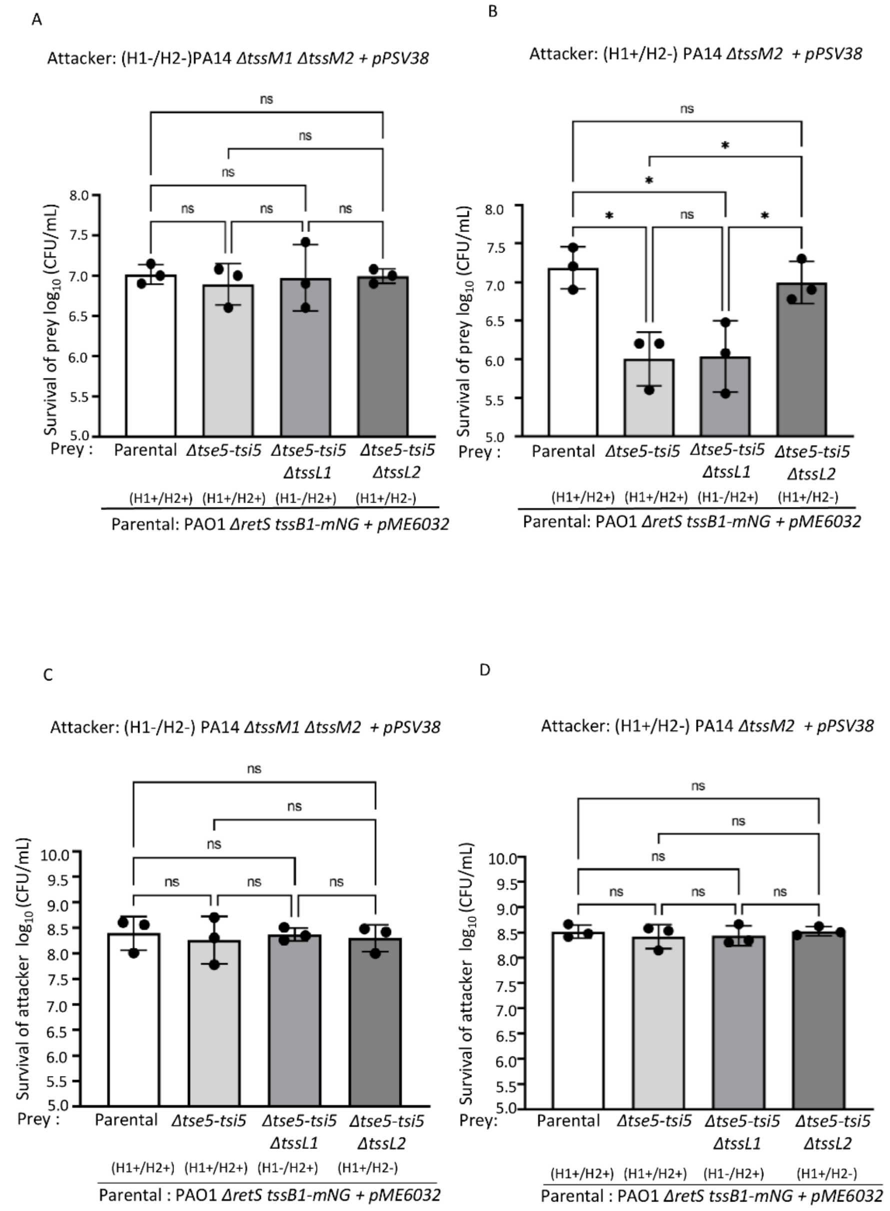

Supplementary Figure 6. Protection of H2-T6SS inactivated non-immune PAO1 prey from PA14 H1-T6SS.

Summary of intraspecific competition assays for H2-T6SS *negative P. aeruginosa* PA14 *attacker* and PAO1 *prey* strains mixed at 20:1 ratio. Data depicted as mean of Log_{10} (CFU/mL) of recovered prey strains after co-incubation with (A) PA14 *ΔretS ΔtssM1 ΔtssM2* attacker and (B) PA14 *ΔretS ΔtssM2* attacker (C) mean of Log_{10}(CFU/mL) of recovered PA14 *ΔretS ΔtssM1 ΔtssM2* attacker, after co-incubation with prey strains (used in Supplementary Figure 6A). (D) Mean of Log_{10} (CFU/mL) of recovered PA14 *ΔretS ΔtssM2* attacker, after co-incubation with prey strains (used in Supplementary Figure 6B). Error bars represent standard deviation of three independent replicates. *p-val<0.1, ns-non-significant; Ordinary one-way ANOVA with multiple comparisons and Tuckey post hoc test.

Supplementary Figure 7

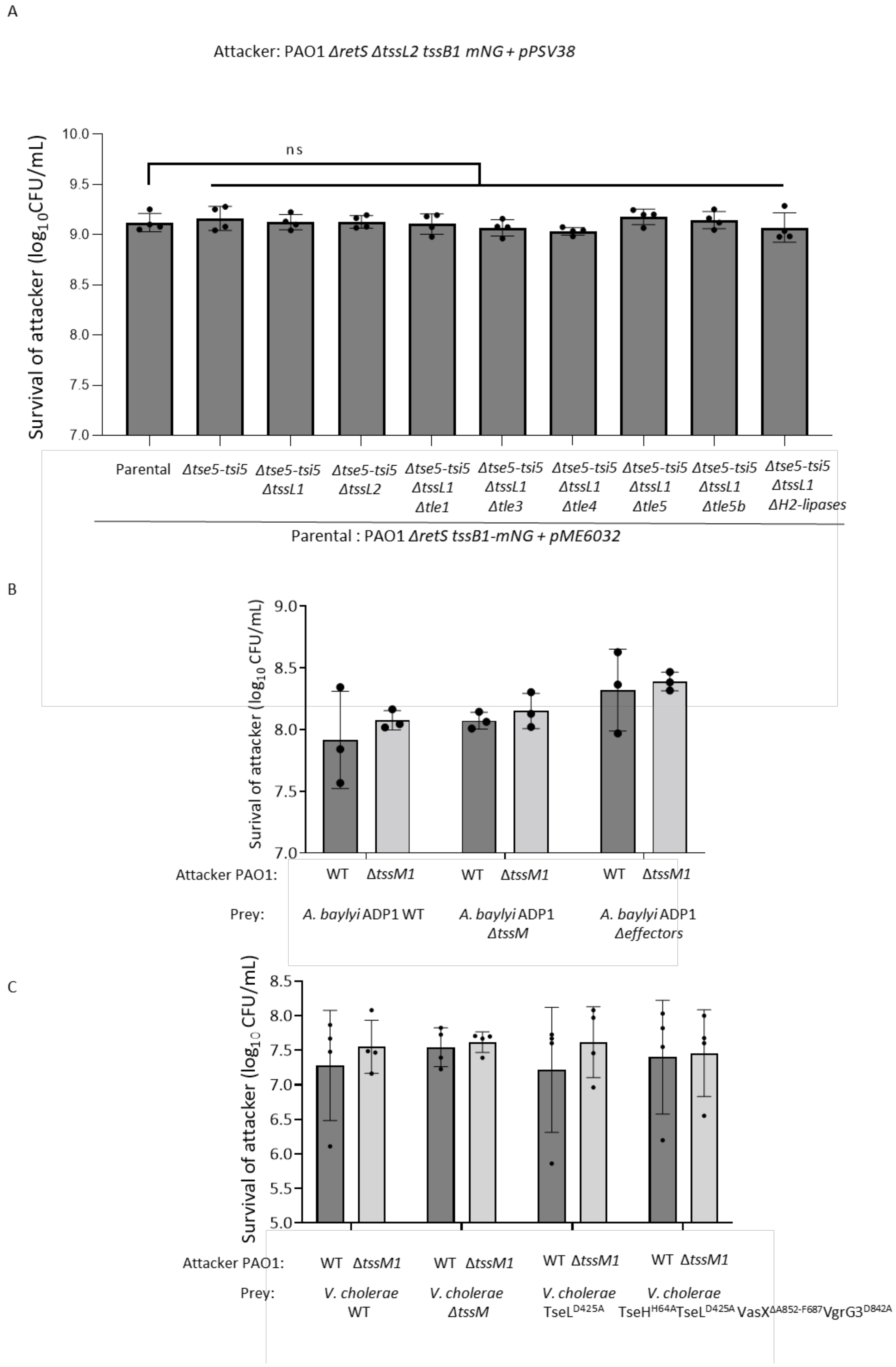

Supplementary Figure 7. Effectors are dispensable for triggering retaliation by the H1-T6SS.

(A) Survival of the attacker *P. aeruginosa* strain when incubated with different (A) *P. aeruginosa,* (B) *A. baylyi* or (C) *V. cholerae* 2740-80 prey strains. Data are presented as mean of Log_{10}(CFU/mL), ns- non-significant; Ordinary one-way ANOVA with multiple comparisons and Tuckey post hoc test.

Table S1: List of strains used in this study

Strain number and description	Source
B129- PAO1, *P. aeruginosa* Irg^{R}, Gent^{S}, Tet^{S}	[1]
B131- PAO1 *ΔretS*	[1]
JS343- PAO1 *ΔretS*, *tssB1-mNG*	This study
JS493- PAO1 *ΔretS*, *tssB2-mCherry2*	[2]
SN696- JS343 + pME6032	This study
SN810- PAO1 *ΔretS*, *ΔtssL1*, *tssB1-mNG*	This study
SN1666- PAO1 *ΔretS*, *Δtse4-tsi4*, *tssB1-mNG*	This study
SN1669- PAO1 *ΔretS*, *ΔPA2778-PA2779*, *tssB1-mNG*	This study
SN1682- PAO1 *ΔretS*, *Δtse2-tsi2*, *tssB1-mNG*	This study
SN1685- PAO1 *ΔretS*, *ΔPA0801-PA0802*, *tssB1-mNG*	This study
SN1688- PAO1 *ΔretS*, *Δtse5-tsi5*, *tssB1-mNG*	This study
SN1692-PAO1 *ΔretS*, *ΔtssL2*, *tssB1-mNG*	This study
SN1695-PAO1 *ΔretS*, *ΔtssL2*, *tssB2-mCherry2*	This study
SN1735-JS343 + pPSV38	This study
SN1736- SN1666 + pME6032	This study
SN1738- SN1669 + pME6032	This study
SN1742- SN1682 + pME6032	This study
SN1744- SN1685 + pME6032	This study
SN1746- SN1688+ pME6032	This study
SN1966- SN1692 + pPSV38	This study
SN2028-SN810 + pPSV38	This study
SN2054- PAO1 *ΔretS*, *Δtse5-tsi5*, *ΔtssL2 tssB1-mNG*	This study
SN2117- PAO1 *ΔretS*, *Δtse3-tsi3*, *tssB1-mNG*	This study
SN2120- PAO1 *ΔretS*, *Δtse6-tsi6*, *tssB1-mNG*	This study
SN2127- SN2117 + pME6032	This study
SN2129- SN2120 + pME6032	This study
SN2132- PAO1 *ΔretS*, *Δtse1-tsi1*, *tssB1-mNG*	This study
SN2134- PAO1 *ΔretS*, *Δtse7-tsi7*, *tssB1-mNG*	This study
SN2146- SN2132 + pME6032	This study
SN2147- SN2134 + pME6032	This study
SN2196- PAO1 *ΔretS*, *ΔtssL1*, *tssB2-mCherry2*	This study

JB105 – *P. aeruginosa* PA14 Irg^R, Gent^S, Tet^S	[3]
sATA112- PAO1 *ΔtssM1*	This study
sATA503 –PA14 *ΔtssM1*	This study
sATA600 –PA14 *ΔretS*	This study
sATA601 –PA14 *ΔtssM1 ΔretS*	This study
sATA751 –PA14 *ΔtssM2*	This study
sATA752 –PA14 *ΔretS ΔtssM2*	This study
sATA753- PA14 *ΔtssM1 ΔtssM2*	This study
SN2230- PA14 *ΔretS, ΔtssM2, tssB1-mNG*	This study
SN2233- SN2054 + pME6032	This study
SN2240- sATA751 + pPSV38	This study
SN2256- sATA753 + pPSV38	This study
SN2306- PAO1 *ΔretS*, *Δtse5-tsi5*, *ΔtssL1, tssB1-mNG*	This study
SN2307- PAO1 *ΔretS*, *ΔtssL2*, *tssB1-mCherry2*	This study
SN2315-PAO1 *ΔretS*, *ΔtssB1*, *clpV2-mNG*	This study
SN2317- SN2306 + pME6032	This study
sATA1061 –PAO1 *ΔretS ΔtssB1 clpV2-mNG ΔtssL2*	This study
sATA945 –PAO1 *ΔretS ΔtssL1 Δtse5-tsi5 tssB1-mNG Δtle5b*	This study
sATA991 –PAO1 *ΔretS ΔtssL1 Δtse5-tsi5 tssB1-mNG Δtle5*	This study
sATA1014 –PAO1 *ΔretS ΔtssL1 Δtse5-tsi5 tssB1-mNG ΔtplE*	This study
sATA1092 –PAO1 *ΔretS ΔtssL1 Δtse5-tsi5 tssB1-mNG Δtle1*	This study
sATA1209 –PAO1 *ΔretS ΔtssL1 Δtse5-tsi5 tssB1-mNG Δtle3*	This study
sATA1213 - PAO1 *ΔretS ΔtssL1 Δtse5-tsi5 tssB1-mNG Δtle5 ΔtplE Δtle5b Δtle1 Δtle3*	This study
sATA1043 –PAO1 *ΔretS ΔtssL1 Δtse5-tsi5 tssB1-mNG Δtle5* + pME6032	This study
sATA1044 –PAO1 *ΔretS ΔtssL1 Δtse5-tsi5 tssB1-mNG ΔtplE* + pME6032	This study
sATA1133 –PAO1 *ΔretS ΔtssL1 Δtse5-tsi5 tssB1-mNG Δtle1* + pME6032	This study
sATA1135 –PAO1 *ΔretS ΔtssL1 Δtse5-tsi5 tssB1-mNG Δtle5b* + pME6032	This study
sATA1215 –PAO1 *ΔretS ΔtssL1 Δtse5-tsi5 tssB1-mNG Δtle3* + pME6032	This study
sATA1216 –PAO1 *ΔretS ΔtssL1 Δtse5-tsi5 tssB1-mNG Δtle5 ΔtplE Δtle1 Δtle5b Δtle3* + pME6032	This study
sATA1214 - PAO1 *ΔretS ΔtssL1 clpV2-mNG Δtle5 ΔtplE Δtle5b Δtle1 Δtle3*	This study
sATA1523 – PAO1 *ΔretS ΔtssL2 tssB1-mNG* + pME6032-TssL2	This study
sATA1524 – PAO1 *ΔtssL2 tssB1-mNG* + pME6032-TssL2	This study
B274- *V. cholerae* 2740-80, lacZ$^-$, Sm^R	[1]

AV005- *Δhcp1*, *Δhcp2*, *V. cholerae* 2740-80, $lacZ^-$, Sm^R	[4]
MI007 - *ΔtssM*, *V. cholerae* 2740-80, $lacZ^-$, Sm^R	[5]
sAP087 - *V. cholerae* 2740-80 $TseL^{D425A}$, $lacZ^-$, Sm^R	This study
sAP195 - *V. cholerae* 2740-80 $TseL^{D425A}$, $VgrG3^{D842A}$, $TseH^{H64A}$ and $VasX^{\Delta A852\text{-}F867}$, $lacZ^-$, Sm^R	This study
JS93 - *V. cholerae* 2740-80, $lacZ^-$, Sm^R, *tssB-mCherry2*	[2]
sAP082.2 - *V. cholerae* 2740-80 $TseL^{D425A}$, $lacZ^-$, Sm^R, *tssB-mCherry2*	This study
B278 - *A. baylyi* ADP1 *rpsL-K88R*, Sm^R	[6]
LLB446 - *A. baylyi* ADP1 *rpsL-K88R*, *tssB-mCherry2*, Sm^R	[7]
sATA694 - A. *baylyi* ADP1 *rpsL-K88R*, Δ*tssM*, *tssB2-mCherry2*, Sm^R	This study
ΔE, A. *baylyi* ADP1 *rpsL-K88R*, Sm^R, effectorless mutant strain	[8]
ΔE, A. *baylyi* ADP1 *rpsL-K88R*, *tssB-mCherry2*, Sm^R	This study

Table S2: List of plasmids used in this study

B30- *E. coli* DH5alpha pEXG2<br>allelic exchange vector with pBR origin, gentamicin resistance, *sacB*	[1]
B130- *E. coli* SM10 pEXG2-*ΔretS_PAO1*	[1]
B202- *E. coli* DH5alpha pWM91<br>allelic exchange vector with oriR6K origin, ampicillin resistance, *sacB*	[9]
B456- *E. coli* SM10 pEXG2-*ΔtssB1_PAO1*	This study
SN259- *E. coli* SM10 pME6032, Tetracycline resistance, replicating vector	[10]
SN2425- *E. coli* DH5alpha pME6032-tssL2	This study
SN914- *E. coli* SM10 pPSV38, gentamicin resistance, replicating vector	[11]
SN797- *E. coli* SM10 pEXG2-*ΔtssL1_PAO1*	This study
Mbr014- *E. coli* SM10 pEXG2-*clpV2-mNG_PAO1*	This study
ML31- *E. coli* SM10 pEXG2-*ΔtssM1_PAO1*	This study
JS240- *E. coli* SM10 pEXG2-*tssB1-mCherry2_PAO1*	[2]
JS341- *E. coli* SM10 pEXG2-*tssB1-mNG_PAO1*	This study
JS59- *E. coli* DH5alpha pWM91-*ΔtssM_V. cholerae* 2740_80	[5]
JS70- *E. coli* DH5alpha pWM91-*tssB-mCherry2_V. cholerae* 2740_80	[2]
JS491- *E. coli* SM10 pEXG2-*tssB2-mCherry2_PAO1*	[2]
SN1600- *E. coli* SM10 pEXG2-*Δtse2-tsi2_PAO1*	This study
SN1601- *E. coli* SM10 pEXG2-*Δtse4-tsi4_PAO1*	This study
SN1602- *E. coli* SM10 pEXG2-*ΔPA0801-PA0802_PAO1*	This study
SN1603- *E. coli* SM10 pEXG2-*ΔPA2778-PA2779_PAO1*	This study
SN1605- *E. coli* SM10 pEXG2-*Δtse5-tsi5_PAO1*	This study
SN2103- *E. coli* SM10 pEXG2-*Δtse3-tsi3_PAO1*	This study
SN2104- *E. coli* SM10 pEXG2-*Δtse6-tsi6_PAO1*	This study
SN2107- *E. coli* SM10 pEXG2-*Δtse1-tsi1_PAO1*	This study
SN2108- *E. coli* SM10 pEXG2-*Δtse7-tsi7_PAO1*	This study
SN1678- *E. coli* SM10 pEXG2-*ΔtssL2_PAO1*	This study
sATA501 – *E. coli* SM10 pEXG2-*ΔtssM1_PA14*	This study
sATA580 – *E. coli* SM10 pEXG2-*tssB1-mNG_PA14*	This study
sATA581 – *E. coli* SM10 pEXG2-*ΔretS_PA14*	This study

sATA747 – *E. coli* SM10 pEXG2-*ΔtssM2_PA14*	This study
SN2401 – *E. coli* SM10 pEXG2-*Δtle5*	This study
SN2402 – *E. coli* SM10 pEXG2-*Δtle5b*	This study
sATA996 – *E. coli* SM10 pEXG2-*ΔtplE*	This study
sATA1076 – *E. coli* SM10 pEXG2-*Δtle1*	This study
sATA1192 – *E. coli* SM10 pEXG2-*Δtle3*	This study
sAP007 – *E. coli* SM10 pWM91-dTseL	This study
sAP012 - *E. coli* SM10 pWM91-dTseH	This study
AV018 - *E. coli* SM10 pWM91-dVgrG3	[4]
AV034 - *E. coli* SM10 pWM91-dVasX	[4]
sAP037 - *E. coli* SM10 pWM91-TseH-H64A	This study
sAP052 - *E. coli* SM10 pWM91-VasX Δ(A852-F867)	This study
sAP053 - *E. coli* SM10 pWM91-VgrG3 D842A	This study
sAP054 - *E. coli* SM10 pWM91-TseL-D425A	This study

<u>**References**</u>

1. Basler, M. & Mekalanos, J. J. Type 6 Secretion Dynamics Within and Between Bacterial Cells. *Science* **337**, 815–815 (2012).

2. Schneider, J. P. *et al.* Diverse roles of TssA-like proteins in the assembly of bacterial type VI secretion systems. *EMBO J.* **38**, e100825 (2019).

3. Rahme, L. G. *et al.* Common virulence factors for bacterial pathogenicity in plants and animals. *Science* **268**, 1899–1902 (1995).

4. Vettiger, A. & Basler, M. Type VI Secretion System Substrates Are Transferred and Reused among Sister Cells. *Cell* **167**, 99-110.e12 (2016).

5. Vettiger, A., Winter, J., Lin, L. & Basler, M. The type VI secretion system sheath assembles at the end distal from the membrane anchor. *Nat. Commun.* **8**, 16088 (2017).

6. Basler, M., Ho, B. T. & Mekalanos, J. J. Tit-for-tat: type VI secretion system counterattack during bacterial cell-cell interactions. *Cell* **152**, 884–894 (2013).

7. Lin, L., Ringel, P. D., Vettiger, A., Dürr, L. & Basler, M. DNA Uptake upon T6SS-Dependent Prey Cell Lysis Induces SOS Response and Reduces Fitness of Acinetobacter baylyi. *Cell Rep.* **29**, 1633-1644.e4 (2019).

8. Ringel, P. D., Hu, D. & Basler, M. The Role of Type VI Secretion System Effectors in Target Cell Lysis and Subsequent Horizontal Gene Transfer. *Cell Rep.* **21**, 3927–3940 (2017).

9. Metcalf, W. W. *et al.* Conditionally replicative and conjugative plasmids carrying lacZ alpha for cloning, mutagenesis, and allele replacement in bacteria. *Plasmid* **35**, 1–13 (1996).

10. Heeb, S. *et al.* Small, stable shuttle vectors based on the minimal pVS1 replicon for use in gram-negative, plant-associated bacteria. *Mol. Plant-Microbe Interact. MPMI* **13**, 232–237 (2000).

11. Yakhnina, A. A., McManus, H. R. & Bernhardt, T. G. The cell wall amidase AmiB is essential for Pseudomonas aeruginosa cell division, drug resistance and viability. *Mol. Microbiol.* **97**, 957–973 (2015).
